# Coronary Heart Disease in Type 2 Diabetes Mellitus: Genetic Factors and Their Mechanisms, Gene-Gene, and Gene-Environment Interactions in the Asian Populations

**DOI:** 10.3390/ijerph19020647

**Published:** 2022-01-06

**Authors:** Khairul Anwar Zarkasi, Nor Azian Abdul Murad, Norfazilah Ahmad, Rahman Jamal, Noraidatulakma Abdullah

**Affiliations:** 1 UKM Medical Molecular Biology Institute (UMBI), Universiti Kebangsaan Malaysia (UKM), Kuala Lumpur 56000, Malaysia; khairul.anwar@upnm.edu.my (K.A.Z.); nor_azian@ppukm.ukm.edu.my (N.A.A.M.); rahmanj@ppukm.ukm.edu.my (R.J.); 2 Biochemistry Unit, Preclinical Department, Faculty of Medicine and Defence Health, Universiti Pertahanan Nasional Malaysia, Kuala Lumpur 57000, Malaysia; 3 Epidemiology and Statistics Unit, Department of Community Health, Faculty of Medicine, Universiti Kebangsaan Malaysia (UKM), Kuala Lumpur 56000, Malaysia; norfazilah@ppukm.ukm.edu.my; 4 Faculty of Health Sciences, Universiti Kebangsaan Malaysia (UKM), Kuala Lumpur 50300, Malaysia

**Keywords:** gene-environment interaction, gene-gene interaction, coronary heart disease, type 2 diabetes mellitus

## Abstract

Asians are more susceptible to type 2 diabetes mellitus (T2D) and its coronary heart disease (CHD) complications than the Western populations, possibly due to genetic factors, higher degrees of obesity, insulin resistance, and endothelial dysfunction that could occur even in healthy individuals. The genetic factors and their mechanisms, along with gene-gene and gene-environment interactions associated with CHD in T2D Asians, are yet to be explored. Therefore, the objectives of this paper were to review the current evidence of genetic factors for CHD, summarize the proposed mechanisms of these genes and how they may associate with CHD risk, and review the gene-gene and gene-environment interactions in T2D Asians with CHD. The genetic factors can be grouped according to their involvement in the energy and lipoprotein metabolism, vascular and endothelial pathology, antioxidation, cell cycle regulation, DNA damage repair, hormonal regulation of glucose metabolism, as well as cytoskeletal function and intracellular transport. Meanwhile, interactions between single nucleotide polymorphisms (SNPs) from different genes, SNPs within a single gene, and genetic interaction with environmental factors including obesity, smoking habit, and hyperlipidemia could modify the gene’s effect on the disease risk. Collectively, these factors illustrate the complexities of CHD in T2D, specifically among Asians.

## 1. Introduction

Diabetes mellitus (DM) is a chronic non-communicable disease that affects approximately 463 million individuals or 9.3% of the total world population [1]. Almost 90% of diabetic patients are classified as type 2 diabetes mellitus (T2D), while others are classified based on their underlying etiology [2]. One of the macrovascular complications in long-term, poorly controlled diabetes is coronary heart disease (CHD) [3,4]. Asia is the most populous continent and home to 60% of global DM cases [5,6]. Although Asians, particularly South Asians, have a lower body mass index (BMI), they exhibit higher degrees of central obesity and total adiposity compared with the matched Caucasian population [7]. Additionally, Asians have higher degrees of insulin resistance and endothelial dysfunction, even among healthy individuals [8]. These factors may contribute to the higher propensity for both T2D and its CHD complications among Asians compared to the Western population, as reported by previous studies [9,10].

The spectrum of CHD includes stable angina and acute coronary syndrome comprising of unstable angina, ST-segment elevation myocardial infarction (STEMI), and non-ST-segment elevation myocardial infarction (NSTEMI) [11]. It is one of the leading causes of morbidity and mortality, resulting in more than 8 million annual deaths worldwide [12]. On top of well-known non-genetic risk factors such as age, gender, family history of CHD, hypertension, dyslipidemia, overweight/obesity and sedentary lifestyle, cigarette smoking, as well as alcohol intake [13,14,15,16,17,18,19,20], various genetic factors have been linked to the disease. Alleles of genetic variants or single nucleotide polymorphisms (SNPs) may influence gene and protein expression by modulating the promoter activity, DNA methylation status, post-transcriptional mRNA processing, mRNA stability, or promoting the interaction with micro-RNA for RNA interference. These could affect certain pathways that are involved in energy and lipoprotein metabolism [18,21], antioxidant mechanisms [22,23], and DNA damage repair [24]. The use of certain drugs for CHD management also has ethnic difference effects between Asians and Caucasians, due to genetic architecture disparity. Antithrombotic clopidogrel for post-MI patients, for example, has a different effect in Asians that had a higher minor allele frequency of the CYP2C19*3 haplotype compared to Caucasians, which was associated with a slower response to clopidogrel therapy [25]. Apart from that, genetic interactions comprising gene-gene and gene-environment interactions, which might modify the risk for CHD and its complications particularly in a T2D background, have been reported in Asian populations. Collectively, the interplay between non-genetic factors, genetic factors, and genetic interactions illustrates the complexities of CHD in T2D.

## 2. Genetic Factors Associated with CHD Risk in T2D Asian Populations

Various studies have shown significant associations of genetic variants with CHD in T2D individuals among Asian populations, as summarized in the next section. These can be divided into several groups depending on their established or proposed roles in the disease mechanism.

### 2.1. Genes Related to Energy and Lipoprotein Metabolisms

The hepatic *APOE* gene encodes apolipoprotein E (apo-E) that is present on the surface of lipoproteins [26,27]. It binds to the hepatic low-density lipoprotein receptor (LDLR) and triggers the internalization of lipoproteins, thus playing an essential role in lipoprotein metabolism [28]. There are three *APOE* gene variants, namely E2, E3, and E4. The E2 variants of the *APOE* gene result in the production of an apo-E protein with an impaired binding affinity towards the LDLR that could contribute to CHD development [29]. A prior study showed that Kuwaiti CHD patients harboring the E2 allele had the highest level of dense LDL particles compared to non-E2 carriers [30]. Besides, plasma apo-E was also higher among individuals with E2 than subjects with E3 allele in the Taiwanese population [31]. Meanwhile, the E4 variant binding affinity toward LDLR was superior to E2 but inferior to E3 [29]. The E4 variant was also an independent predictor for both T2D and cardiovascular disease (CVD) among the Egyptians. It was associated with a 5.90-fold higher risk for having CVD and T2D compared to other *APOE* variants in this population [32]. Contrarily, the E3 variant is the neutral form of *APOE* and has the strongest binding affinity to LDLR [29]. Individuals who carry this variant exhibit higher levels of high-density lipoprotein (HDL), higher antioxidant protein expressions and β-carotene levels, as well as lower blood low-density lipoprotein (LDL) concentrations [33].

**Figure 1:**
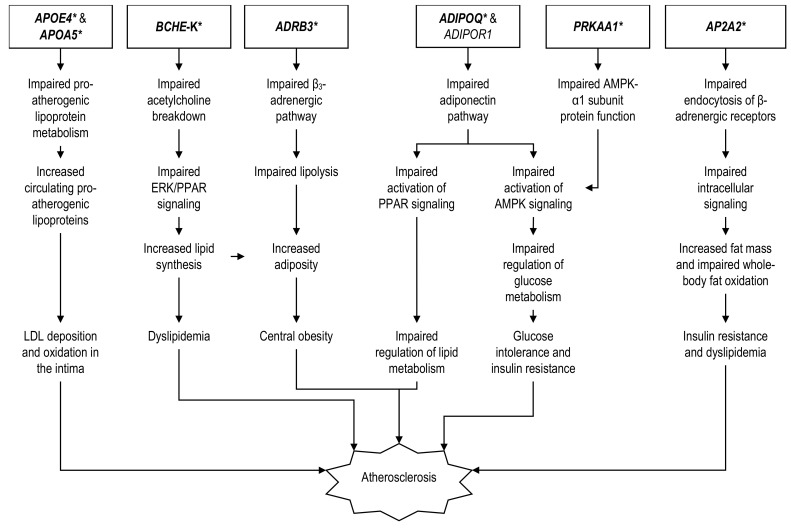
Proposed mechanisms of genes related to energy and lipoprotein metabolisms associated with CHD in the T2D Asian population. * Significant association with CHD exclusive in T2D Asians but not T2D Caucasians. Abbreviations: AMPK (5′ adenosine monophosphate-activated protein kinase), ERK (extracellular signal-regulated kinase), LDL (low-density lipoprotein), PPAR (peroxisome proliferation-activated receptor).

Apart from its function in lipoprotein metabolism, the apo-E protein could also regulate the arterial wall composition via the matrix metalloproteinase-9 (MMP-9) enzyme to maintain arterial compliance. Apo-E-deficient mice exhibited fragmentation of elastin laminae in their aortae, which was closely related to MMP-9 activity [34]. Compared to the apo-E3 protein, apo-E4 is more prone to proteolysis. As such, the administration of fragmented or proteolyzed apo-E4 upregulated MMP-9, which, in turn, activated elastin fragmentation in human astrocytoma and neuroblastoma cell lines [35]. Since elastin laminae run parallel to collagen fibers in the arterial wall, the fracture of elastin caused stress to be transferred directly to the stiffer collagen leading to vascular stiffening and subsequent hypertension [34]. The process appeared to precede the formation of atherosclerotic plaque, which was in agreement with the study by Weiss et al., (2001), who reported the exacerbation of atherosclerosis by the simultaneous presence of apo-E deficiency and hypertension in mice [34,36]. Furthermore, apo-E had been shown to affect glucose metabolism in mice astrocytes. Compared to E2/E3, astrocytes expressing the E4 allele were associated with reduced glucose uptake, increased anaerobic glycolysis, and increased gluconeogenesis [37], which might result in hyperglycemia. Concerning lipoprotein metabolism, the E4 allele could also influence HDL formation. Mice expressing the hepatic *APOE4* gene exhibited enhanced VLDL ability to bind lipids along with impaired lipolysis and reduced VLDL remnants clearance compared to mice with hepatic *APOE3* [38]. Subsequently, there would be decreased VLDL surface components availability that could be transferred to the HDL pool for HDL production, resulting in reduced plasma HDL concentrations and increased pro-atherogenic lipoproteins levels (Figure 1) [38].

*APOE* gene expression is the highest within the liver, followed by the brain and other tissues, including the heart [39]. In the animal model, targeted replacement of the *APOE* gene to generate mice with the E4 allele established clear T2D phenotypes at basal conditions [40]. These mice displayed reduced insulin secretion, insulin resistance, and glucose intolerance [40]. In humans, variation of the *APOE* gene, specifically the E4 allele, could exert a significant CHD risk in T2D Asian patients. A study by Vaisi-Raygani et al. (2010) reported that the Iranian T2D carriers of the E4 risk allele had a significantly higher CHD risk in the allelic (odds ratio, OR: 2.30), dominant (OR: 2.17), and recessive models (OR: 7.80) (Table 1) [41]. They also observed that E4 carriers had significantly higher serum total cholesterol (TC), triglyceride (TG), and LDL, alongside lower serum HDL concentrations [41]. Another independent study among the Thai population reported a similar finding in which the T2D carriers of the E4 allele were associated with a 2.32-fold higher CHD risk [18]. Although the recessive genotype E4/E4 did not have a significant association with CHD, the E3/E4 genotype still conferred a markedly 2.52-fold higher CHD risk in this population [18]. Similar to the Iranian population, the E4 allele is associated with an impaired fasting lipid profile including increased VLDL and TG, as well as decreased HDL in T2D patients with or without CHD [18]. Subjects in the Asian Indian Diabetic Heart Study/Sikh Diabetes Study showed no differences in the frequency of the E4 allele nor its genotypes in either CHD or T2D patients compared to non-CHD or non-T2D controls [42]. Nevertheless, individuals with the E4 allele still possessed higher CHD risk factors, including elevated systolic (SBP) and diastolic blood pressure (DBP), fasting glucose, and postprandial glucose, as well as lower HDL levels than non-E4 carriers [42]. Despite the significant association between the E4 allele and increased CHD risk among Caucasians in Italy, Finland, Australia, and the United States [43,44,45,46], the association was absent when analyzed specifically among T2D Caucasian patients [47]. Moreover, a cross-sectional study among 271 T2D Finnish patients showed that the prevalence of CHD was similar between the E4 (64.4%) and E3 phenotypes (54.5%, *p* > 0.05) [48], indicating that E4 was not a CHD risk allele among T2D individuals in this population.

Another gene closely related to *APOE* is *APOA5*, which encodes for the apolipoprotein A5, a protein that associates with TG-rich lipoproteins and is present on the HDL surface [74,75]. The gene is expressed specifically by the liver [76]. Apo-A5 could bind to the LDL receptor and may have a role in lipoprotein metabolism [77]. Additionally, apo-A5 could activate the lipoprotein lipase enzyme and promote TG breakdown [74]. Mice fed with a high fructose diet developed signs of T2D, which include insulin resistance as measured by hyperinsulinemic-euglycemic clamps and elevated fasting insulin level [78]. These changes were associated with elevated *ApoA5* mRNA and ApoA5 protein expression in the liver, increased hepatic and skeletal muscle lipid deposition, as well as abnormal lipid profiles that were reversed by an antisense oligonucleotide to suppress the gene expression [78]. One of the many *APOA5* gene polymorphisms associated with CHD is −1131T/C. In a South Indian cohort, the −1131C risk allele frequency was significantly higher among the T2D + CHD subjects compared to non-T2D + CHD and healthy controls [22]. It translated to a 1.71-fold higher CHD risk among the T2D patients than the non-diabetic controls, as well as a 1.50-fold higher CHD risk among the T2D patients compared to healthy individuals [22]. However, the study team did not assess the association of the *APOA5* risk allele with other clinical parameters in this population [22]. Compared to Indian Asians, European Caucasian subjects had a significantly lower frequency of −1131C regardless of their T2D status [79,80]. Despite the difference, the variant effect on the plasma TG level was similar in both populations [79]. The *APOA5* −1131C exerted a two- to three-fold increased CHD risk among general Caucasians in Hungary and Czech with a similar direction seen among Asians [81,82]. However, these studies did not examine the −1131C effect on CHD risk in the T2D subgroup [81,82].

Upon catecholamine stimulation, excessive heart contraction is prevented by the β_3_-adrenergic receptor, which exerts negative inotropic effects on the myocardium [83]. Activation of the receptor by a β_3_-agonist nebivolol was shown to be beneficial in the myocardial infarct (MI) animal model, potentially by supporting myocardial activity via promoting lipolysis as the primary cardiac energy source, increasing energy usage, while decreasing body adiposity [83,84]. *ADRB3* is another gene linked to T2D and coronary heart disease wherein *ADRB3* polymorphism (Trp64Arg) is associated with central obesity, dyslipidemia, and insulin resistance, all of which are known risk factors for CHD [85]. Type 2 diabetes induction in C57BL/6J mice by a high-fat diet resulted in increased plasma insulin, increased blood glucose levels, as well as reduced *GLUT4* mRNA expression indicating impaired plasma glucose clearance [86]. These changes corresponded to decreased *Adrb3* mRNA expression in the white adipose tissue causing central obesity, in which the mice accumulated perirenal, retroperitoneal, mesenteric, and uterine adipose tissues [86].

In a human study conducted among T2D Hong Kong Chinese, Wang et al. (2010) reported that individuals with the homozygous arginine (Arg/Arg) *ADRB3* genotype had a 3.84-fold higher hazard for CHD incidence under a recessive model [21]. The study did not analyze the association of the *ADRB3* risk genotype with other clinical variables [21]. Meanwhile, a recent meta-analysis investigating the *ADRB3* gene Trp64Arg polymorphism showed that carriers of the mutated 64Arg allele consistently and significantly had higher CHD risk compared to subjects harboring the wild-type 64Trp allele, with the allelic (OR: 1.48), additive (OR: 2.66), and recessive models (OR: 2.46) [87]. However, the association of 64Arg with CHD was significant only in individuals of Asian but not Caucasian descent, and analysis regarding the 64Arg effects on clinical variables was not performed in this meta-analysis [87]. In agreement with this, several bodies of evidence showed that there was no association between *ADRB3* 64Arg with T2D and CHD in Caucasians. In a population-based cohort of 1259 Germans, the levels of plasma TC, HDL, fasting insulin, HbA_1c_, BMI, as well as T2D status did not differ among different Trp64Arg genotypes [88]. In the same manner, 64Arg did not correlate with incident CHD among Caucasians from various countries including The Netherlands, United Kingdom, Germany, United States, and Finland either in the dominant, recessive, or additive models [89].

Butyrylcholinesterase (BChE) is an enzyme that belongs to the cholinesterase family. It metabolizes short-acting neuromuscular blockers such as mivacurium and succinylcholine, in addition to acetylcholine and other choline esters [90]. Additionally known as pseudocholinesterase, the enzyme is encoded by the *BCHE* gene. The impact of T2D on BCHE protein activities had been reported in previous animal experiments. For instance, Dave and Katyare (2002) observed that alloxan-induced diabetes in rats significantly increased the plasma and cardiac BCHE activities, which could not be reversed by insulin therapy [91]. On the other note, mice with *BCHE* gene deficiency developed central obesity and an impaired lipid profile, as well as early signs of the T2D phenotype characterized by marginally elevated FBG and significantly elevated fasting insulin levels [92]. Meanwhile, in a human study, Asian individuals carrying the *BCHE* K-variant of the gene that harbored a 1615A polymorphism (Ala539Thr) were significantly associated with a 2.02-fold increased risk for CHD among T2D patients under an allelic model [41]. The T2D + CHD subjects harboring *BCHE* 1615A also had numerically higher serum TG, TC, and LDL, but similar HDL levels compared to those without the risk allele [41]. However, there was no statistical test conducted to assess differences between the two groups [41]. In Caucasians, conflicting evidence regarding *BCHE* K-variant association with T2D exist. In the United Kingdom, the K-variant lacks association with the necessity for insulin therapy despite being positively correlated with T2D status [93]. A study by Johansen et al. (2004) observed a similar allelic frequency of the *BCHE* K-variant between T2D and control subjects among 1320 Danish Caucasians [94]. Likewise, all *BCHE* genotypes exhibited comparable BMI as well as serum insulin, C-peptide, FBG, and 2-h plasma glucose levels following an oral glucose tolerance test (OGTT) [94]. In terms of CHD risk, Italian Caucasians had comparable K-variant frequencies in both CHD and control groups [95], indicating that the gene did not contribute much to CHD in this population.

Although the exact mechanism on how it leads to CHD is unknown, *BCHE* was proposed to heighten the disease risk by altering the lipid parameters and body adiposity. Butyrylcholinesterase is produced by the hepatocytes, enters the circulation, and promotes ghrelin production by enteroendocrine cells known as ghrelin cells in the gastrointestinal mucosa [96,97]. As a gut–brain hormone, ghrelin travels from the gastrointestinal system to the brain, binds to the ghrelin receptor in the lateral hypothalamus, then stimulates the hunger center [98]. Mice deficient in the *BChE* gene exhibited increased plasma ghrelin compared to wildtype. Increased plasma ghrelin elevates appetite, resulting in increased daily calorie intake, body weight, fat mass, and plasma leptin concentration, as well as decreased daily energy expenditure, which was reversed upon intravenous *BChE* gene transfer [92,99]. Besides, specific inhibition of *BChE* had been shown to increase the levels of acetylcholine, whereas acetylcholine could increase lipid synthesis via the nicotinic acetylcholine receptor and upregulate ERK/PPAR-γ signaling [100,101]. In contrast to the animal experiment, a study in human subjects reported that individuals with increased serum BChE correlated significantly with overweight and obesity [102]. This association was significant for all body adiposity surrogate markers including BMI (OR: 1.16), waist circumference (OR: 1.07), waist–hip ratio (OR: 16.90), and waist-height ratio (OR: 4.51) [102]. Human subjects with higher BChE levels were more likely to have elevated serum TG, TC, and LDL, along with decreased HDL, compared to those with lower BChE levels, indicating that BChE could regulate lipid metabolism [102].

Adiponectin is a substance encoded by the *ADIPOQ* gene that is specially produced and released by the fatty tissues, with adipocyte size and adipose mass influencing its blood concentration [103,104,105]. Meta-analyses of human studies reported that weight loss by caloric restriction, intake of weight-loss medications such as sibutramine, and bariatric surgery significantly increased the adiponectin level [106,107,108]. In the liver and skeletal muscle, adiponectin regulates lipid and glucose metabolisms by improving glucose uptake, reducing gluconeogenesis, glycogenolysis, and lipid content [109]. Indeed, *Adipoq* deficiency in mice was capable of inducing diabetic characteristics such as an impaired glucose tolerance test, increased blood glucose, free fatty acids, as well as TG levels [110]. In the same study, there was also a reduction of the pancreatic islet size, β-cell mass, and blood insulin level in *Adipoq^–/–^*mice compared to WT [110]. Additionally, adiponectin could exert antioxidative and anti-inflammatory effects by reducing inducible nitric oxide synthase (iNOS) as well as promoting macrophage tolerance to interleukin 6 (IL-6) and tumor necrosis factor-alpha (TNF-α) stimulation in the vascular endothelium [111,112]. Adiponectin could play a significant role in alleviating atherosclerosis by decreasing LDL oxidation, improving cholesterol efflux from macrophages, and enhancing endothelial functions [113,114,115]. Monocytic migration and smooth muscle cell proliferation in the intimal layer of the artery are also prevented by adiponectin [116,117].

Concerning its anti-atherosclerotic effects, a low level of adiponectin had been reported to have a strong association with an increased CHD risk, which could also be influenced by the *ADIPOQ* gene polymorphism [118,119,120]. A prior study reported that the GG genotype of the *ADIPOQ* G276T variant (rs1501299) was associated with lower serum adiponectin compared to the 276TT genotype [121]. This variant was also related to an increased CHD risk in T2D, especially in obese individuals. Katakami et al. (2012) reported that among 2637 adult Japanese T2D patients, the risk of CHD was increased by 1.49 times for each number of the 276G allele, while those having 276GG genotype had 1.66 times higher CHD risk compared to 276GT + TT genotypes [56]. They also found that the recessive 276GG genotype associated with a slightly higher DBP measurement (G/G: 79.0 ± 10.0 mmHg; G/T + T/T: 78.5 ± 10.5 mmHg, *p* = 0.0218), while the presence of the 276G risk allele corresponded to lower serum adiponectin levels (G/G: 6.11 ± 3.69 μg/mL; G/T: 6.74 ± 4.92 μg/mL; T/T: 9.56 ± 9.21 μg/mL, *p >* 0.05) [56].

In line with that, Esteghamati et al. (2012) observed that the 276T risk allele provided a protective effect against CHD in North Iranian T2D patients with an OR of 0.39 despite the allele having no impact on the serum adiponectin levels (*p* = 0.48) [13]. However, in another study among the Southwest Iranian T2D population, individuals with the 276TT genotype had a 5.16-fold increased CHD risk compared to the 276GG homozygotes [65], suggesting that other contributing factors could interact with the genetic polymorphism in different ancestries. Even though baseline characteristics of CHD patients showed significantly lower serum adiponectin, an abnormal lipid profile, and elevated BP compared to healthy controls, a comparison of clinical parameters for each genotype was not performed [65]. Meanwhile, a study of 11 common variants for the *ADIPOQ* gene in a T2D Chinese population found that –11377C/G polymorphism (rs266729) was associated with both CHD status and coronary atherosclerosis severity on angiography [58]. The risk for CHD was 2.18 times, 1.53 times, and 1.49 times for the recessive (–11377GG), co-dominant (–11377CG), and allelic models (–11377G), respectively [58]. Similar to 276G, the –11377G variant had a significant negative association with serum adiponectin levels (β = –0.101) in this study [58]. The CHD risk among T2D was also increased when there was *ADIPOQ* 45T/G polymorphism (rs2241766). Among 224 Iranian T2D patients undergoing coronary angiography, the odds of having CHD were 7.21 in the 45GG compared to 45TT, although analysis of the differences in clinical parameters among genotypes were not performed in this study population [64]. In contrast, a separate study that was also conducted among T2D Iranians reported no association of the *ADIPOQ* 45G variant with CHD risk nor the serum adiponectin level [13], indicating the possibility of genetic effect modification by environmental factors. Concerning Caucasians, *ADIPOQ* 276T functioned as a CHD risk allele for non-diabetic, but a protective allele against CHD for diabetic patients [122,123]. Unlike Asians, *ADIPOQ* 45G was not associated with CHD risk at all [122,123]. As for *ADIPOQ* –11377G, positive associations could only be seen with unstable angina and CHD, but not with T2D among Russian Caucasians [124], suggesting that –11377G is a risk allele for CHD in T2D subjects in Asians but not Caucasians.

Adiponectin binds to the adiponectin receptors, AdipoR1 and AdipoR2, and triggers signaling cascades such as the 5′ adenosine monophosphate-activated protein kinase (AMPK) and the peroxisome proliferation-activated receptor (PPAR) in the target cells [125]. The *ADIPOR1* gene encodes AdipoR1. It is ubiquitously expressed but can be found more in muscle cells, including cardiomyocytes [126]. In contrast, the AdipoR2 expression is restricted to the liver [103]. The action of adiponectin is highly dependent on its receptors. Decreased AdipoR1 and AdipoR2 expression in the liver of *db/db* mice, an animal model of human obesity, exhibited diminished adiponectin-induced AMPK and PPAR activation resulting in glucose intolerance as well as reduced insulin sensitivity [127]. Apart from that, diabetic status caused deregulation of the *AdipoR1* gene in the animal model. Streptozotocin (STZ)-induced DM in mice was associated with a significant increase of *AdipoR1* mRNA expression in the skeletal muscle, which reverted to normal levels upon insulin treatment [128]. Similarly, *AdipoR1* mRNA was also increased in the skeletal muscle of T2D *db/db* obese and *Lepr^–/–^*mice, in which the overexpression of *AdipoR1* prevented diabetes by improving insulin sensitivity [127,128]. On the other hand, *AdipoR1* deficiency in mice impaired normal cardiac physiology in which there was reduced myocardial mitochondrial function and cardiac inefficiencies, which were inapparent in mice deficient with *AdipoR2* [129].

In this regard, common *ADIPOR1* gene variants have been implicated with CHD in non-diabetic Asian subjects [63,67]. Likewise, the gene also has a significant association with CHD in T2D. Among the Northeast Chinese population, three out of six *ADIPOR1* variants were markedly associated with a higher CHD risk. They include rs3737884*G (OR: 2.69), rs16850797*C (OR: 1.44), and rs7514221*C (OR: 1.75) [67]. However, there was no assessment of the genotypic impact on clinical variables performed in the study [67]. In a separate study among a North Chinese population, T2D individuals with *ADIPOR1* variants rs3737884*G (OR: 2.42) and rs16850797*C (OR: 1.71) had a significantly higher CHD risk as well [63]. Both variants highly significantly correlated with increased BMI, SBP, DBP, fasting blood glucose (FBG), TG, and TC, as well as decreased HDL levels [63]. Like Asians, past publications indicated that the *ADIPOR1* gene was significantly associated with T2D and CHD among Caucasians, although the variants involved differed. *ADIPOR1* variants relevant to elevated T2D risk in Caucasians include rs2275737*T and rs1342387*G [130], whereas rs7539542*G and rs10920531*A conferred an increased CHD risk among Caucasians with T2D comorbidity [131]. The latter two variants were associated with decreased *ADIPOR1* mRNA expression in the study populations as well [131].

The activation of AdipoR by adiponectin activates the AMPK signaling pathway that regulates carbohydrate and lipid metabolism [109]. Adiponectin could also prevent atherogenesis and endothelial dysfunction via the AMPK pathway [132]. The AMPK is a heterotrimeric protein complex comprised of a catalytic α-subunit, and regulatory β- and γ-subunits [133]. Previous studies reported that the α1 isomer of the catalytic α-subunit exerted protective effects against diabetes, endothelial dysfunction, and atherosclerosis. Overexpression of AMPK-α1 in the liver ameliorated hepatic steatosis in hyperlipidemic T2D rats [134], while *Prkaa1*-specific deletion in the mAMPKα1-KO mice muscle resulted in T2D characteristics including hyperglycemia, hyperlipidemia, and increased muscle TG contents when they were fed a high-fat diet [135]. Conversely, selective deletion of the protein kinase AMP-activated catalytic subunit α1 (*Prkaa1*) gene that encodes for AMPK-α1 in endothelial cells resulted in reduced endothelial cell proliferation as well as enhanced atherosclerotic formation in mice with hyperlipidemia [136]. Meanwhile, a study on T2D patients of Northern China reported a negative association between the *PRKAA1* rs3805489*C variant with CHD. Among 404 participants with T2D, the rs3805489*C allele carriers had 0.67 times less CHD risk compared to the A allele carriers [60]. Additionally, the dominant rs3805489*AC + CC genotype also had protective effects against CHD in this study population (OR: 0.65) [60]. However, they did not assess the impact of the rs3805489*C risk allele on clinical parameters in the study population [60]. At present, there is no study regarding *PRKAA1* polymorphism association with CHD and T2D in Caucasians.

The *AP2A2* gene encodes the adaptor-related protein complex 2 (AP-2) α2 subunit. The AP-2α2 protein has a functional role in the clathrin-mediated endocytosis, where it is localized to the clathrin-coated pit plasma membrane [137]. It mediates endocytosis of various proteins on the cell surface, including glucose transporter-4 (GLUT-4), the insulin receptor, and the β-adrenergic receptor, in which the endocytosis process is required for active signaling intracellularly [138]. *AP2A2* is also one of the target genes for PPAR-α whose activity promotes fat mass loss and whole-body fat oxidation. Mice with adipocyte-specific *Ap2a2* deficiency exhibited impaired endocytosis of the β_2_- and β_3_-adrenergic receptors, as well as reduced cyclic adenosine monophosphate and protein kinase A (cAMP/PKA) signaling, resulting in larger fat mass and decreased whole-body fat catabolism when they were fed with a high-fat diet [138]. However, the impact of *Ap2a2* deficiency on glycemic control was minimal with no overt T2D phenotype developed in the mice [138]. The AP-2 protein had been shown to interact with several genes such as *NUMB* in the mouse intestine, resulting in decreased dietary cholesterol uptake; as well as with *DAB2* and *LDLRAP1* associated with pronounced hypercholesterolemia [139], indicating its essential role in lipid metabolism. Meanwhile, the T allele of rs7396366, whose closest gene is *AP2A2*, had an association with increased plasma C-reactive protein (CRP), which is a biomarker in acute CHD and endothelial dysfunction [140,141]. Therefore, individuals with *AP2A2* genetic variation might be susceptible to T2D and CHD, although the mechanism on how it influences disease development apart from altering lipid metabolism and promoting obesity is unknown. Indeed, among T2D Chinese patients, *AP2A2* rs7396366*T carriers had a 2.33-fold raised CHD risk for rs7396366*GT + TT genotypes compared to rs7396366*GG homozygotes [73]. Furthermore, the *AP2A2* rs7396366 polymorphism is associated with more severe CHD, although its association with other clinical parameters was not determined [73]. T2D patients with rs7396366*TT had 2.37 times and 2.48 times higher probability for two-vessel and three-vessel diseases, respectively, compared to the rs7396366*GG genotype [73]. There is no evidence regarding the association of the *AP2A2* gene, specifically the rs7396366 polymorphism, with T2D and CHD among Caucasians since studies are yet to be performed in this population.

### 2.2. Genes Related to Vascular and Endothelial Pathology

**Figure 2:**
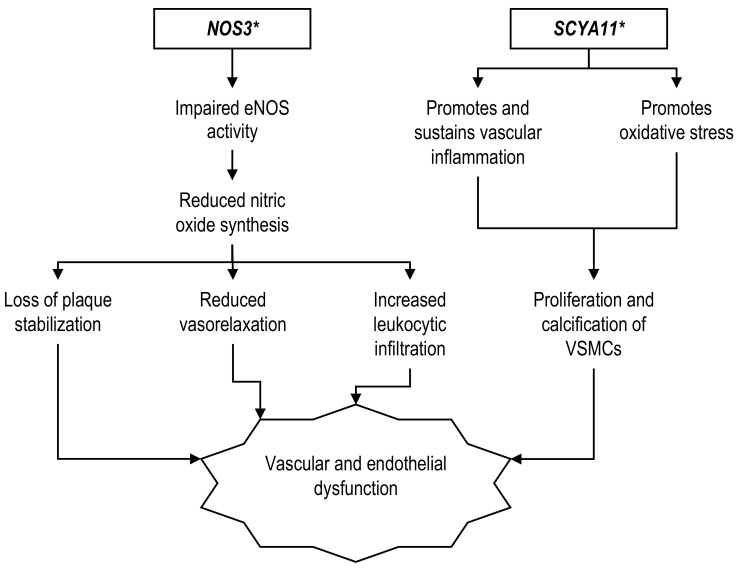
Proposed mechanisms of genes related to vascular and endothelial pathology associated with CHD in the T2D Asian population. * Significant association with CHD exclusive in T2D Asians but not T2D Caucasians. Abbreviations: eNOS (endothelial nitric oxide synthase), VSMCs (vascular smooth muscle cells).

The human eotaxin is encoded by the *SCYA11* gene, also known as *CCL11* [142]. Eotaxin is expressed by smooth muscle cells as well as immune cells such as macrophages and lymphocytes [143]. It was proposed that eotaxin promoted and sustained vascular inflammation by triggering oxidative stress along with proliferation and calcification of vascular smooth muscle cells (VSMCs), which was implicated in atherogenesis (Figure 2) [144]. The atheromatous plaque contains an abundant amount of human eotaxin that functions as a chemoattractant, inducing VSMCs migration [145]. Hessner et al., (2004) reported increased *SCYA11* gene expression in the pancreatic islets and lymph nodes of autoimmune insulin-dependent DR*lyp/lyp* rats, the animal model of human type 1 diabetes mellitus [146]. These changes were associated with β-cell damage and insulitis compared to the WT control [146]. Similarly, feeding C57BL/6N mice a high-fat diet induced obesity [147], which is a strong risk factor for T2D. Although the effects of insulin resistance and glycemic control were not examined, the study observed a significant increase in adipose eotaxin mRNA and serum eotaxin concentration [147]. Nevertheless, no animal model can justify T2D development by the *SCYA11* mutation at present. A genetic study among a 1386 Hong Kong Chinese T2D cohort observed that the Ala allele carriers of the *SCYA11* Ala23Thr variant were associated with a higher incidence of CHD in both the additive (hazard ratio, HR: 1.49) and recessive models (HR: 1.70) [21]. However, no assessment of the risk allele association with other clinical variables was conducted in this population. The 23Ala risk allele appeared to be causative only in the Asian populations. Studies conducted among American and Czech subjects found that the opposite 23Thr allele was associated with higher odds and incident CHD; however, these studies were performed on general CHD patients and not specifically among T2D subjects [142,148]. Nevertheless, Caucasian CHD patients with the Ala/Thr + Ala/Ala genotype were older and exhibited higher proportions of hyperlipidemia as well as diabetes compared to CHD patients with the Thr/Thr genotype, although these were statistically insignificant [148].

The endothelial nitric oxide synthase, encoded by the *eNOS*/*NOS3* gene, is expressed constitutively by the endothelium. It generates nitric oxide (NO) that promotes vasodilatation, inhibits leukocytic infiltration, and prevents smooth muscle cell migration and proliferation [149]. Mice with permanent left anterior descending (LAD) coronary artery ligation had a reduced ejection fraction of 40–46% when implanted with vascularized cardiac spheroids (VCS) generated from co-culturing *eNOS* gene knocked-out cardiomyocytes and endothelial cells [150]. Conversely, the ejection fraction was higher (61%) when implanted with VCS generated from WT cardiomyocytes and endothelial cells co-culture [150]. Furthermore, the administration of an NO donor, molsidomine, resulted in atherosclerotic plaque stabilization, cardiac function improvement, and MI reduction in the MI mice model compared to the control, which demonstrated the cardioprotective effects of NO [151]. Meanwhile, rats that spontaneously developed T2D had changes in the expression level of islet *eNOS* mRNA associated with oxidative stress and inflammation [152]. Besides, mice with *eNOS* deficiency developed overt T2D when fed a high-fat diet, while those who received standard chow had hyperinsulinemia and limited insulin resistance with a normoglycemic background [153]. These changes associated with disturbed insulin signaling in the coronary endothelial cells resulted in coronary vascular dysfunction [153].

Observations from animal models displayed the *eNOS* association with cardiac pathology and T2D. In fact, the association could also be observed in a human epidemiological study. The *eNOS* –786T/C gene polymorphism was associated with a higher risk of T2D among Korean subjects, with –786C being the risk allele [52]. The same study also reported that the variant was markedly associated with a 4.39-times-higher CHD risk among T2D individuals harboring –786TC + CC compared to –786TT genotypes [52]. However, the study lacked further analysis of the genotypic effect on other clinical parameters [52]. Consistent with this result, Narne et al. (2013) observed that South Indian T2D patients carrying the –786C risk allele had 1.81-fold increased CHD risk in a dominant inheritance model [59]. Besides, the dominant model also significantly correlated with increasing CHD severity especially the triple vessel disease (OR: 2.99) [59]. When assessing the variant association with other parameters, the study reported that subjects with *eNOS* –786C were more likely to have BMI more than 25 kg/m^2^ (OR: 2.00), positive family history of T2D (OR: 1.99), hypertension (OR: 1.90), and being an active smoker (OR: 2.81) [59]. A similar result was also reported for the *eNOS* 4a/4b variant. The 4a/4b + 4a/4a is significantly associated with 4.20-fold higher CHD risk in T2D Asians compared to 4b/4b genotypes [52], despite having no association with other clinical variables [59]. Another *eNOS* gene G894T variant did not appear to have an association with CHD when tested among T2D patients of both the Northern and Southern Indian regions [57,59]. Nonetheless, non-diabetic (but not diabetic) CHD patients with the 894T-containing genotype had a correspondingly lower serum NO level than those with the 894T-non-containing genotype, measured at 5.44 ± 3.24 mmol/L and 12.13 ± 7.21 mmol/L, respectively [57].

By comparison, a case-control study involving 1197 Australian Caucasians did not find any differences in terms of allelic and genotypic frequencies of *eNOS* –786T/C, 4a/4b, or G894T between CHD and healthy community controls, either in both or separate genders [154]. The lack of association between the three *eNOS* gene polymorphisms was also evident in a prospective cohort among Caucasians in the United Kingdom [155]. The risk of a CHD event after 8.1 years of follow-up failed to show any association with –786T, 4a, or 894T alleles, additionally with no effect on plasma NO regardless of cigarette smoking status [155]. However, both studies reported their findings from general CHD subjects but did not perform subgroup analysis for CHD with a T2D background. Evidence also showed that the *eNOS* gene variant only had a significant association with clinical parameters, such as increased SBP and mean arterial pressure in Finnish Caucasians, particularly when they had CHD and T2D comorbidity, but there was no direct association between *eNOS* and the individual diseases [156].

### 2.3. Genes Related to Antioxidation

**Figure 3:**
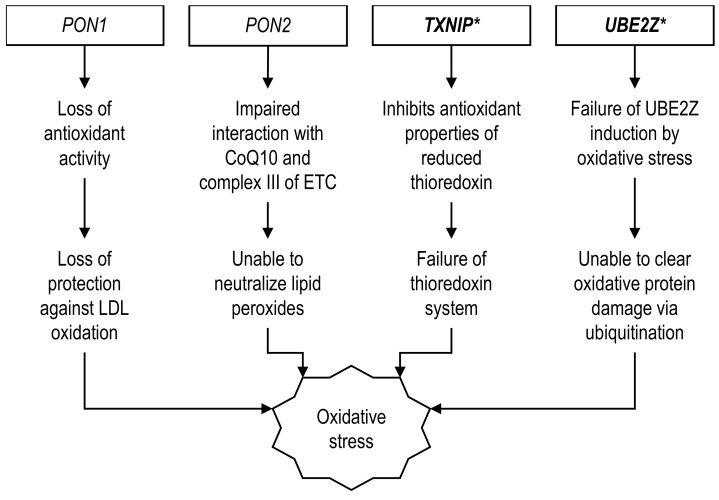
Proposed mechanisms of genes related to antioxidation mechanisms associated with CHD in the T2D Asian population. * Significant association with CHD exclusive in T2D Asians but not T2D Caucasians. Abbreviations: CoQ10 (coenzymeQ10 or ubiquinone), ETC (electron transport chain).

Paraoxonase (PON) is an esterase enzyme comprising three variants, namely PON-1, PON-2, and PON-3 [157]. PON-1 and PON-3 are present on HDL particles and enter general circulation, whereas PON-2 is ubiquitously present within many cell types. Nevertheless, they share almost similar antioxidant properties, protecting cells, and preventing LDL particles against oxidative damage [157]. The association of the *PON1* gene with oxidative stress and diabetes had been reported in a T2D animal model. In a study by Koren-Gluzer et al. (2013), *PON1*-knockout (KO) mice that received a high-fat diet developed increased oxidative stress and abnormal insulin signaling causing marked impairment on a glucose and insulin tolerance test as well as impaired whole-body glucose uptake, which was contributed by decreased muscle GLUT-4 protein expression [158]. The administration of exogenous PON-1 to mice before STZ-injection conferred beneficial effects. It prevented the formation of diabetes characteristics by attenuating STZ-induced serum glucose elevation and serum insulin reduction [159]. The mechanism through which PON-1 protected against diabetes was promoting β-cell viability, increasing β-cell’s insulin content, and promoting insulin release from β-cell [159]. On the other hand, the Q192R polymorphism of the *PON1* gene resulted in the mutation of arginine for glutamine on the 192nd position of the PON-1 protein. In the South Indian population with T2D, this resulted in a 1.49-fold increased risk for CHD among the 192R risk allele carriers [22]. Asian diabetic patients with CHD who also had the 192RR genotype were also shown to have decreased PON-1 enzyme activity, suggesting that the enzyme function was negatively affected by the Q192R polymorphism causing the disease [54]. Since LDL oxidation is one of the earliest steps in atherogenesis, the Q192R PON-1 protein variant would have lost its antioxidant activity and failed to protect LDL from being oxidized, promoting plaque formation (Figure 3). Another *PON1* gene polymorphism, L55M, triggered the replacement of methionine in place of leucine at position 55. However, the 55L risk variant did not appear to significantly affect the PON-1 enzyme functions in T2D Asian populations as it was not associated with increased CHD risk, disease severity, or alter the FBG level [49]. Unlike Asians, where 192RR was associated with decreased PON-1 activity, the 192RR genotype was linked to increased PON-1 activity in T2D Czech Caucasians [160]. It was also significantly associated with better diabetic control in T2D patients as well as lower blood glucose level in CHD patients [160,161]. Regardless, Caucasians who carried the *PON1* 192R allele still had a higher risk of CHD and CHD severity, measured at 1.78-fold for CHD and 1.92-fold for the three-vessel disease [162].

One of the sources of reactive oxygen species (ROS) in CHD comes from mitochondria. Myocardial hypoxia following coronary blockage would cause electron leakages due to reduced function of the electron transport chain (ETC) complexes, particularly complexes I and III, which would result in ROS, such as superoxide anions (O_2_^●–^) [163,164,165]. A prior study had shown that PON-2 localized in the inner mitochondrial membrane, associated with complex III, and interacted with coenzyme Q10 (ubiquinone), a mobile electron carrier in the ETC [166]. *PON2*-deficient mice developed significant atherosclerotic lesions in the aorta, which was associated with decreased complex I and III activities, reduced ATP levels, and increased superoxides in the hepatic mitochondria [166]. Meanwhile, functional analyses using HeLa cells found that overexpression of the human *PON2* gene protected the cells against mitochondrial dysfunction by improving ATP production as well as lowering mitochondrial superoxide levels [166]. Similar to *PON1*, the *PON2* gene could also contribute to the T2D phenotype in the animal model. When fed an obesogenic diet, *PON2*-KO mice were reported to have adipocyte hypertrophy resulting in increased fat mass and body weight [167]. Correspondingly, these mice also had elevated fasting insulin and impaired tolerance to glucose, but the plasma lipid profile remained unchanged [167].

Point mutation of the *PON2* gene, in which amino acid cysteine substitutes serine at position 311 (Ser311Cys), decreases total serum PON levels in human subjects [168]. Reduced serum PON may affect its antioxidant properties, promoting disease development. Certainly, the Ser311Cys variant of the *PON2* gene was related to increased CHD incidence in Asians with diabetes. Among 1386 T2D Hong Kong Chinese, the mutated 311Cys allele conferred an elevated risk for CHD in both the additive (HR: 1.42) and dominant models (HR: 1.55) [21]. Nevertheless, the study did not measure the 311Cys risk allele effect on serum PON or oxidative stress levels in this population [21]. These results replicated the findings among Caucasians. Like Asians, the role of 311Cys on CHD development is particularly more important in diabetic than non-diabetic individuals. Italian Caucasians with a dominant *PON2* 311Cys genotype with a past history of acute MI were associated with T2D, lower HDL, and higher troponin T levels [169].

Another endogenous antioxidant present in the human body is the thioredoxin (TRX) system comprising the TRX protein, TRX reductase (TRXR), and NADPH as its core components [170]. Reduced TRX molecules could scavenge free radicals by donating electrons in the form of hydrogen molecules while being oxidized during the process [170]. Subsequently, the oxidized TRX would be transformed back into a reduced state by the action of TRXR. A binding protein called the TRX-interacting protein (TXNIP) regulates the TRX system by inhibiting the antioxidation properties of a reduced TRX rendering it inactive [171]. In a disease state, mutation of the TXNIP gene could aggravate the TXNIP inhibitory activity on the reduced TRX, delay free radical scavenging, and increase oxidative stress. *Txnip*-KO mice had improved insulin sensitivity, in which insulin-dependent glucose uptake was improved by 36% and 40% in the skeletal muscle and white adipose tissue, respectively [172]. Additionally, *Txnip*-deficient mice were also resistant to diabetes induction by STZ injection. Upon STZ administration by the standard protocol, Hcb-19/*Txnip*^–/–^mice had preserved plasma insulin and pancreatic insulin content, as well as a lower plasma blood glucose concentration compared to the control group [173]. These findings were correlated with higher β-cell mass, higher pancreatic islet count and size, as well as a lower degree of β-cell apoptosis [173]. Meanwhile, the administration of a high-fat diet in *Txnip*-KO mice, which mimics human T2D pathogenesis more, did not prevent increased adiposity or increased plasma TG and FFA [172]. However, these mice were protected against T2D phenotypes of hyperglycemia and hyperinsulinemia due to improved insulin sensitivity and glucose absorption in the skeletal muscle and adipose tissues [172]. In the endothelial cells, *Txnip* could promote vascular inflammation by inhibiting the expression of Krupple-like factor 2, an anti-inflammatory transcription factor [174]. Using human umbilical vein endothelial cells (HUVEC) and a flow apparatus, regions with turbulent flow showed a significant increase of pro-inflammatory mRNA expression including vascular cell adhesion molecule-1 (*VCAM1*) and intercellular adhesion molecule-1 (*ICAM1*), which were reversed by *Txnip* gene deletion [174].

In parallel to that, a previous human study reported that Asian CHD subjects carrying the *TXNIP* rs7212*G variant had elevated malondialdehyde (MDA) levels, a marker of lipid peroxidation [68], confirming its role in oxidative stress. Evidence showed that *TXNIP* polymorphism also correlated with increased CHD surrogate markers, including arterial stiffness and abnormal glucose metabolism, as well as increased CHD risk. T2D Chinese patients with the CG + GG genotype of rs7212 had a 1.53-times higher chance of developing CHD compared to the CC homozygotes in a dominant model [68]. Moreover, both dominant (CG + GG) and recessive models (GG) of rs7212 are associated with elevated *TXNIP* mRNA expression and plasma MDA levels [68], confirming the occurrence of oxidative damage by the gene polymorphism. Other *TXNIP* variants, such as rs7211 and rs9245, did not seem to be associated with CHD status in a diabetic background, although the former did show a significant association with higher CHD risk in a general non-diabetic Chinese population but not for the latter [68]. At present, no study has examined the association between the *TXNIP* gene, particularly rs7212, and CHD in Caucasians. Nonetheless, Connelly et al., (2014) observed an increase of the *TXNIP* gene and protein expression from the right atrial tissue obtained from T2D Americans undergoing cardiac surgery [175]. The level of right atrial *TXNIP* mRNA was elevated more than 30-fold, while TXNIP protein was increased by 17%, which was associated with a 21% reduction of TRX protein activity compared to the non-diabetic group [175]. These findings serve as evidence regarding a plausible *TXNIP* role for future CHD complications in T2D Caucasians.

Ubiquitination is a four-step process that transfers a ubiquitin group to a target protein for proteasomal degradation. Ubiquitin-conjugating enzyme E2 Z (UBE2Z) mainly plays a role in the second step of ubiquitination [176]. An in vitro study demonstrated that hydrogen peroxide (H_2_O_2_)-induced oxidative stress in HeLa cells triggered gene transcription of various ubiquitin-conjugating enzymes, including *Ube2z* to tag misfolded proteins and form protein aggregates for autophagic clearance [177]. The possible role of the *Ube2z* gene in T2D pathogenesis is strengthened further by Dreja et al. (2009) [178]. In this study, New Zealand mice fed a high-fat diet developed signs of T2D characterized by hyperglycemia and hyperinsulinemia [178]. A genome-wide scan revealed more than 2109 differentially expressed genes in the diabetic mice pancreas, including *Ube2z* [178]. Recent human studies also showed that the *UBE2Z* might play a role in the development of hyperglycemia and hyperlipidemia, both of which are strongly associated with oxidative stress. The *UBE2Z* gene expression was increased in the pancreatic samples of T2D subjects, in addition to a strong positive correlation with increased TG and LDL concentrations [179,180,181]. These three parameters are known risk factors for CHD. As of now, no animal model could justify *Ube2z* mutation leading to CHD. Despite the lack of evidence, transcriptome analysis revealed that *Ube2z* mRNA transcripts were present within the cardiac endothelial cells [182], which might serve some function in the pathogenesis of CHD.

Considering its molecular role in oxidative stress, mutation of *UBE2Z* may also impact CHD development, especially with diabetes as a background in Asian individuals. Indeed, Lu et al., (2017) reported that *UBE2Z* rs46522*T polymorphism increased CHD risk among North Chinese T2D patients, in which the association could be seen in the additive (OR: 1.67) and recessive models (OR: 1.28) [70]. Nonetheless, the gene’s effects on other clinical parameters or its detailed functional role in the disease process are still yet to be elucidated. Findings in the Asians replicated what has been observed in a large genome-wide association (GWA) study of more than 140,000 European descents [183]. The rs46522*T mapped to *UBE2Z* was associated with 1.06-fold increased CHD risk that reached genome-wide significance, with a *p*-value of 1.81 × 10^−8^ [183]. Despite positive findings between *UBE2Z* and CHD, evidence linking the gene to T2D in Caucasians is not supportive. In an Icelandic population, rs46522*T failed to show any correlation with T2D status [184]. It also did not correlate with blood pressure, lipid profile, plasma HbA_1c_, glucose, and insulin levels, as well as central obesity measured by fat mass percentage [184].

### 2.4. Genes Related to Cell Cycle Regulation

The cyclin-dependent kinase 2A (*CDKN2A*), *CDKN2B*, and *MTAP* genes are located at 9p21 [185]. The *CDKN2A* encodes p14^ARF^ and p16^INK4a^, whereas *CDKN2B* codes for p15^INK4b^, all of which take part in cell-cycle regulation. Meanwhile, the *MTAP* gene encodes the methylthioadenosine phosphorylase enzyme that plays a role in polyamine biosynthesis [186]. The role of 9p21 in T2D development had been shown in animal models. For instance, mice with *Cdkn2a* gene deficiency were protected against high-fat diet-induced obesity, had higher energy expenditure, and had better insulin sensitivity compared to *Cdkn2a*-normal mice [187]. Human T2D is closely related to the aging process. Apart from causing insulin resistance, evidence showed that *Cdkn2a* (or more specifically p16^INK4a^) also protected against age-related pancreatic function decline [188]. Deficiency of the gene in *p16^–/–^* mice promoted β-cell survival, which was associated with higher pancreatic islet proliferation in old mice compared to the WT group [188]. p16^INK4a^ is also an endothelial progenitor cellular senescent marker, whereas polyamines have multi-functionalities including oxidative stress scavengers, regulators of DNA expression and amino acid synthesis, as well as promoters for cellular growth and proliferation [189,190]. Thus, these proteins might contribute to CHD by enhancing cellular senescence along with reduced smooth muscle cell proliferation leading to plaque instability. Additionally, a long non-coding RNA, ANRIL, is also encoded by the *CDKN2A/2B* gene cluster in the antisense direction [191]. ANRIL had been shown to increase inflammatory cytokine levels (e.g., IL-6, IL-8, vascular endothelial growth factor, and TNF-α) associated with an endothelial injury that supported atherogenesis [192,193].

Single nucleotide polymorphisms of the 9p21 locus showed significant correlations with CHD in T2D Asian populations. In a study by Cheng et al. (2011), two 9p21 SNPs responsible for T2D and CHD were rs10811661 and rs10757283 [53]. The T allele carriers of rs10811661 had 1.23- and 1.19-times higher risk for T2D and CHD, respectively, than healthy controls [53]. For the C allele carriers of rs10757283, the risk was 1.30 times for T2D and 1.18 times for CHD compared to the control group, with an additional positive association with CHD severity (β = 0.267, *p* = 0.002) [53]. Correspondingly, other separate studies among T2D Chinese reported that the rs944801*C risk allele and rs10757274*AG + GG genotypes conferred 1.10 times and 4.38 times significantly higher risk for MI, respectively [62,194]. However, the risk alleles’ impact on other clinical variables was not explored in all of these studies. From the four 9p21 SNPs mentioned previously, only rs944801*C and rs10811661*T had a positive association with CHD and T2D among Caucasians, similar to findings in T2D Asians [72,195,196,197]. On the other hand, rs10757274*G had a positive association with CHD in general Caucasians but lacked association with T2D markers such as fasting glucose, insulin, and HbA_1c_ [198,199]. Meanwhile, a previous publication reported that rs10757283*T conferred a 1.10-times increased T2D risk in a large GWA study of 898,130 European ancestries [200]. Despite this strong positive association, no evidence could link either the T or C allele of rs10757283 with CHD in Caucasians.

The 9p21 locus could also be useful as a marker for CHD severity. Wang et al. (2010) reported that carriers of the rs1333049*C risk allele had a more severe atherosclerotic plaque progression in a dose-dependent manner, which was measured as changes in the minimum luminal diameter, changes in the diameter of stenosis, coronary artery score, and cumulative coronary obstruction [50]. In this study, statistical significance was seen only among non-T2D + CHD patients, but a similar pattern could also be observed among T2D + CHD subjects [50]. The extent of stenosis indicating disease severity could also be calculated using the Gensini score upon coronary angiography [201]. Individuals in the fourth quartile of the Gensini score carrying a mutated C allele of 9p21 rs10757283 had a two-fold higher CHD risk compared to those in the first quartile [53].

### 2.5. Genes Related to DNA Damage Repair

Poly(ADP-ribose) polymerase 1 (PARP-1) is involved during the first process of DNA damage detection in the base excision repair (BER) process for single-strand breaks (SSB). They bind to the damaged site and undergo autoribosylation to generate poly(ADP-ribose) on themselves as well as on other proteins (e.g., histones) in the SSB vicinity. The poly(ADP-ribose) would serve as a marker in initiating the next steps of the BER process [202]. Despite its importance in preserving DNA integrity, an in vitro study showed that hyperactivation of PARP-1 in the HUVECs led to nicotinamide dinucleotide (NAD^+^) and ATP depletion with subsequent cell death and apoptosis [203]. Inhibition of the PARP-1 significantly improved the ischemia-reperfusion injury in rat cardiomyocytes as well as preventing cardiac complications in the diabetic rat model [204,205]. Besides, mice with *Parp1*-deficiency were protected against the perturbation of glucose metabolism such as hyperglycemia and hyperinsulinemia following STZ-induced DM [206]. On the other hand, Devalaraja-Narashimha et al., (2010) observed that *Parp1*-KO mice were metabolically less active and more susceptible to T2D characteristics including obesity, fatty tissue accumulation, insulin resistance, hyperleptinemia, and glucose intolerance following high-fat diet consumption compared to the WT group [207]. Findings from the latter study could be explained by PARP-1’s important role in supporting initial adipocyte differentiation from stem cells to preadipocytes and adipocyte lineages, as well as during terminal differentiation into mature adipocytes [208]. Less-differentiated adipocytes display marked lipodystrophy [209]. Thus, PARP-1 deficiency might partly contribute to T2D by causing glucose and lipid homeostasis impairment in the adipose tissue.

Meanwhile, human epidemiological studies particularly among Asians noted significant associations of *PARP1* gene polymorphism with T2D and CHD. For example, Wang et al. (2017) reported that PARP-1 activity was not only elevated in Chinese CHD patients compared to healthy controls; it also positively correlated with the CHD severity [71]. In this population, PARP-1 protein activity was influenced by the *PARP1* rs1136410 polymorphism, with the lowest levels seen in the GG genotype and the highest levels observed among AA genotypes [71]. As such, CHD risk among the G allele carriers of rs1136410 was lower compared to non-carriers in a recessive model (OR: 0.73) that corresponded to decreasing PARP activities with an increasing number of rs1136410*G allele [71]. In terms of the lipid profile, rs1136410*G allele had a marginal negative correlation with serum TC (β = −0.096, *p* = 0.081) and a significant positive correlation with serum HDL (β = 0.026, *p* = 0.002) [71]. Contrarily, the opposite result was obtained when sub-analysis was performed among T2D Chinese subjects, whereby the GG genotype of rs1136410 had a significant increase of CHD risk by 1.02-fold compared to the AG + GG genotype (*p* = 0.01) [71]. This finding marked a significant gene–environment interaction leading to the modification of the *PARP1* gene effect on CHD. Conversely, the association between *PARP1* rs1136410 with CHD in T2D Caucasians could not be established since the study in this population is lacking.

### 2.6. Genes Related to Hormonal Regulation of Glucose Metabolism

Proprotein convertase 1, also known as prohormone convertase 3 (PC 1/3), encoded by the proprotein convertase subtilisin/Kexin type 1 (*PCSK1*) gene, plays an essential role in the initial step of insulin and glucagon-like peptide 1 (GLP-1) synthesis. It converts pro-insulin in the pancreatic β-cell and pro-glucagon in the intestinal L-cell into insulin and GLP-1, respectively. GLP-1 exerts incretin effects via GLP-1 receptor stimulation that triggers downstream signaling cascades in the pancreas [210]. In contrast to the hyperglycemic actions of the pancreatic glucagon, intestinal GLP-1 promotes glucose-stimulated insulin secretion by β-cells while decreasing glucagon release by α-cells [210]. Apart from regulating glucose metabolism, insulin may exert atheroprotection by suppressing oxidative stress, inflammatory signaling, and leukocytic infiltration, as well as improving the lipid profile [211]. Complimentarily to insulin activity, GLP-1 could prevent atherosclerosis by preserving the endothelial function while reducing small dense LDL, oxidized LDL, and foam cell formation [212]. Previous studies in animal models confirmed the *PCSK1* gene’s plausible role in T2D development. Neonatal mice with homozygous null *Pc1/3* exhibited decreased hepatic *IGF1* mRNA and protein expression, as well as growth retardation, whereas prolonged glycemic response was only observed in heterozygous mice [213]. Another study by Lloyd et al. (2006) discovered that *Pc1* N222D polymorphism in mice was associated with impaired processing of proinsulin, in which there was reduced mature insulin production despite normal proinsulin synthesis, suggesting abnormal Pc1 protein activity [214]. The *Pc1*^N222D/N222D^ mice also exhibited adipocyte hypertrophy, increased fat content with elevated total body weight, and impaired glucose tolerance test scores compared to *Pc1^+/+^* [214]. These changes signified T2D risk, although the mutant mice did not develop overt hyperglycemia in this study [214].

Genetic variants involving *PCSK1* in humans might lead to decreased PC 1/3 protein synthesis and activity, with additional association with impaired fasting glucose, obesity, as well as a deranged lipid profile, including TG and HDL [215]. These effects might explain how the *PCSK1* variants could lead to CHD in T2D Asian populations. Among 683 Chinese with T2D, the rs156019*T variant was associated with an increased CHD risk under additive (AT vs. TT, OR: 1.92) and dominant inheritance models (OR: 1.76) [61]. In contrast, protection against CHD in T2D might be conferred by the rs3811951*G allele, which showed reduced odds of having CHD at 0.75 times, 0.64 times, and 0.43 times compared to the rs3811951*A allele in the respective allelic, additive, and recessive manners [61]. Other *PCSK1* gene variants, including rs6230, rs6233, and rs6234, did not appear to influence CHD development among T2D subjects in this population [61]. Apart from these findings, the influence on other clinical data by rs156019 and rs3811951 polymorphisms was not explored in this study [61]. At present, no study assessed *PCSK1* rs156019, rs3811951, rs6230, rs6233, and rs6234 variants’ impact on CHD risk among T2D or non-diabetic Caucasians. Nevertheless, rs6234 was reportedly in linkage disequilibrium with rs6235, which was shown to be associated with a 1.02 elevated risk for T2D [216]. The variant was also positively correlated with 2-h plasma glucose, 2-h plasma insulin, HbA_1c_ levels, and BMI among subjects with European ancestry [216].

The binding of insulin to the insulin receptor triggers conformational changes, autophosphorylation of its β-domain, and activation of insulin signaling cascades. One of the various effects of insulin signaling is the translocation of GLUT-4 to the plasma membrane that mediates glucose entry into the cells [217]. The *ENPP1* gene encodes a protein regulator of insulin signaling called the ectonucleotide pyrophosphatase/phosphodiesterase-1 protein. The binding of ENPP-1 to the α-domain of the insulin receptor prevents the activation of insulin signaling with subsequent GLUT-4 storage resulting in insulin resistance and hyperglycemia that could result in DM [217]. The effects could be observed in an animal model as reported by Pan et al. (2011) [218]. Targeted *ENPP1* overexpression in mice adipocytes when they were exposed to high-fat food caused changes that were relevant and similar to human T2D [218]. These include decreased insulin receptor phosphorylation rendering them inactive, with subsequent development of fatty liver, increased hepatic TG content, an impaired plasma lipid profile, hyperglycemia, hyperinsulinemia, as well as impaired glucose and insulin tolerance test scores [218]. The *ENPP1* might also have a direct role in neointimal progression. Silencing of the gene with small interfering RNA (siRNA) exhibited a 90% reduction of ENPP-1 protein levels associated with 1–3-fold and 3–5-fold enhanced proliferation of human VSMCs and human induced pluripotent stem cells (iPSC)-derived VSMCs, respectively [219].

In human studies, the presence of *ENPP1* rs1044498*C polymorphism in Caucasian populations is associated with insulin resistance when assessed by the homeostasis model assessment (HOMA-IR), accompanied by elevated HbA_1c_, fasting insulin, and fasting plasma glucose levels [220]. At the same time, a meta-analysis of nine case-control studies involving Caucasians discovered that the *ENPP1* rs1044498*C variant was significantly associated with increased CHD in allelic (OR: 1.25) and dominant models (OR: 1.16) [221]. Similarly, a Taiwanese population harboring *ENPP1* rs1044498*C was related to central obesity, high fasting glucose, and T2D [222]. Correspondingly, T2D South Indian patients who harbored the polymorphic AC + CC genotypes of rs1044498 had a significantly enhanced risk for CHD compared to AA homozygotes (OR: 12.8) [69]. This mutation causes a single amino acid change from lysine to glutamine at the 121st position of ENPP-1 protein (K121Q), leading to a gain-of-function mutation. The mutation could lead to a higher degree of insulin resistance, T2D, as well as diabetic vascular complications such as CHD, as seen in these epidemiological studies. However, the effects of the rs1044498*C risk allele on ENPP-1 protein activity as well as metabolic and clinical parameters remained unclear as it was not assessed in this population [69].

### 2.7. Genes Related to Cytoskeletal Function and Intracellular Transport

The KIF6 gene encodes kinesin-like protein 6, a member of the kinesin-9 family under the kinesin superfamily. It is considered as the molecular motor, taking part in intracellular microtubular-dependent transport of organelles, mRNAs, and proteins [223]. One of the few *KIF6* variants widely studied is 719Arg (rs20455). Not only is the variant associated with decreased HDL and increased TG, but it has also been shown to be significantly associated with the risk of developing non-fatal MI [224,225]. Although KIF-6 is not directly involved in the regulation of lipid metabolism, prior studies reported that carriers of the *KIF6* 719Arg variant benefited more from statin therapy than non-carriers [226,227]. Due to its significant association with lipid levels, the response to lipid-lowering medication, and non-fatal MI, the 719Arg might also be linked to CHD development in T2D individuals. Wu et al., (2014) identified that the same variant was associated with a 5-fold higher CHD risk in T2D in a dominant model of 719Trp/Arg + Arg/Arg compared to the 719Trp/Trp genotype (OR: 5.21) [17]. In line with previous reports, higher serum TG and lower serum HDL were noted in the T2D and T2D + CHD carriers of the 719Arg risk allele [17]. Comparatively, *KIF6* 719Arg was significantly associated with increased CHD and MI risk as well as future coronary events among general Caucasians [228,229]. Apart from that, we could not find any studies that examined the association between 719Arg with T2D or T2D + CHD in this population.

The association of *KIF6* with lipid parameters and CHD risk in T2D was only reported in human epidemiological studies. In a previous animal study by Hameed et al. (2013), *Kif6* ΔE3 mutant mice, which mimic 719Arg in humans, had increasing serum TC and TG levels for every ΔE3 allele, but the differences were statistically insignificant [230]. Serial cardiac function tests also appeared similar in both homozygous *Kif6*^ΔE3/ΔE3^ and heterozygous *Kif6*^ΔE3/+^ mice compared to WT *Kif6*^+/+^ [230]. On the same note, no animal model can justify T2D development following *KIF6* gene mutation at present. *KIF6* mRNA and proteins are highly expressed in the brain, lungs, and reproductive tissues according to the Human Protein Atlas database [231]. Meanwhile, the gastrointestinal tract, hepatic, cardiac, adipose, as well as pancreatic tissues express a lower amount of *KIF6* mRNA but lack KIF-6 protein expression [231]. Thus, the mechanism on how the *KIF6* gene variant causes CHD in T2D subjects may require further exploration.

Histone deacetylase 9 (HDAC9) has a role in the histone deacetylation process with a subsequent influence on gene transcription. Shroff et al., (2019) reported that subjects carrying the *HDAC9* rs2107595 risk allele had 155 differentially expressed genes involved in the regulation of β-cellular gene expression, the diabetes pathway, IL-6 signaling, cholesterol efflux, and platelet aggregation that might have an impact on T2D development and its CHD complication [232]. Together with HDAC4 and HDAC5, HDAC9 has been observed to regulate pancreatic growth and development [233]. As reported by Lenoir et al. (2011), mice lacking the *Hdac9* gene (*Hdac9^–/–^*) exhibited increased insulin-producing β-cell mass in the pancreas compared to WT [233]. In parallel to this finding, chronic high-fat diet consumption led to impaired adipocyte differentiation to a mature state, which was associated with increased *Hdac9* gene expression [234]. The mice also displayed T2D characteristics of increased weight gain, insulin resistance, glucose intolerance, and hepatosteatosis, which were ameliorated in *Hdac9*-KO mice [234].

Increased *HDAC9* gene expression could also result in increased calcification and decreased contractility of the human aortic smooth muscle cells, while *HDAC9* deficiency in mice resulted in 40% less aortic calcification and a better survival rate than wildtype [235]. The gene also appeared to take part in the atherosclerotic process wherein systemic and bone marrow cell-specific *HDAC9* deletion in *LDLr*-deficient mice led to depressed cholesterol efflux and macrophage activation, the upregulation of genes involved in lipid homeostasis, as well as the downregulation of inflammatory genes [236]. A previous study reported that *HDAC9* deficiency in mice prevented neointimal formation induced by arterial ligation, providing further evidence of the gene’s contribution toward atherosclerosis and CHD [237]. Nurnberg et al. (2020) found that *HDAC9* co-localized with *TWIST1*, a gene that has a functional role in modulating the phenotypes of smooth muscle cells, in the human aorta samples [238]. *TWIST1* knock-down in the human coronary artery smooth muscle cells produced less-differentiated cells with a more proliferative nature [238]. Therefore, the mechanism through which *HDAC9* might cause atherosclerosis and CHD may be by influencing the *TWIST1* gene activity.

In regard to the strong association of the gene with atherosclerosis, the *HDAC9* rs2107595*A variant also showed increased CHD risk by 1.61-fold in a T2D Chinese population for AG + AA compared to GG genotypes [66]. In a dominant model, the variant was strongly correlated with higher *HDAC9* mRNA and protein expression in the MI subgroup, as well as associated with more severe coronary atherosclerosis as determined by the Gensini score [66]. Moreover, overweight/obese, diabetic, and hyperlipidemic patients of this population also had significantly higher Gensini scores when they dominantly carried the rs2107595*A risk allele [66]. In Caucasians, the same *HDAC9* rs2107595*A variant had a positive association with CHD status as reported in two large European-based GWA studies [239,240]. However, these studies did not examine the *HDAC9* rs2107595*A association with CHD in the T2D subgroup. Moreover, despite strong evidence of *HDAC9*’s role in the development of T2D in animal models, the association between *HDAC9*, particularly rs2107595 polymorphism, with T2D in both Asian and Caucasian subjects has not been reported so far.

## 3. Genetic Interaction Associated with CHD Risk in T2D Asians Populations

### 3.1. Gene-Gene Interaction

Gene-gene interaction, or epistasis, is a phenomenon where the effect of one gene could modify the effect of another gene or several genes on a disease phenotype [241]. Gene-gene interaction might modify the patient’s phenotype by enhancing or reducing the risk to develop complex diseases such as CHD and T2D. Methods to assess gene–gene interaction include regression and generalized multifactor dimensionality reduction (GMDR). Regression analysis is relatively simple and commonly used, but it tends to have an overfitting problem especially in high-order interaction, elevated type I and II errors, as well as decreased robustness due to sparse data in the multifactorial model [242,243]. Meanwhile, GMDR employs a machine-learning strategy with enhanced capability to test for high-order interactions and handle large-scale data as seen in GWA studies [243]. In this review, most of the gene-gene interactions reported in Asian populations were analyzed using regression techniques.

The *BCHE*-K and *APOE* genes, which are related to energy and lipoprotein metabolisms, could influence CHD development in T2D by altering lipid parameters [42,102], as well as promoting obesity, as shown in previous studies [102,244]. Individually, both genes exerted a similar two-fold increased CHD risk in Iranian T2D patients. The presence of risk variants for both genes was associated with markedly higher serum LDL, TG, and TC, as well as lower serum HDL levels. These altered lipid profiles accompanied a significant doubling of CHD risk in this population (OR: 4.50, *p* = 0.011) when both genetic risk variants were present (Table 2) [41].

In CHD pathogenesis, the roles of *ADRB3*, *SCYA11*, and *PON2* gene variants, which are related to energy and lipoprotein metabolisms, vascular and endothelial pathology, and antioxidant mechanisms, respectively, appeared to be complementary. The *ADRB3* variant may increase CHD-modifiable risk factors as it correlates with central obesity and dyslipidemia [85]. On the other hand, the *SCYA11* variant may support atherogenesis, while polymorphism of *PON2* may reduce its antioxidation capability leading to oxidative stress [144,157]. By using recessive models for *SCYA11* Ala23Thr and *ADRB3* Trp64Arg, as well as an additive model for *PON2* Ser311Cys, Wang et al. (2010) analyzed their association with cardiac endpoints within an 8-year follow-up period. Considering individuals that carry ≤1 risk allele as a reference, the hazard for developing cardiac endpoints (defined as the occurrence of CHD or heart failure) increased steadily to 2-fold for two risk alleles carriers (HR: 1.99, *p* = 0.026), almost 3-fold for three risk alleles (HR: 2.74, *p* = 0.003), and 4-fold for four risk alleles carriers (HR: 4.11, *p* = 0.002) [21].

Oxidative stress is a common observation in CHD. Mechanisms that promote oxidative stress include an imbalance in the antioxidant and pro-oxidant enzyme activities. The *GCLM* and *SOD2* are both parts of the antioxidation machinery. While *GCLM* encodes the modifier subunit of glutamate-cysteine ligase, an enzyme involved in the glutathione-producing pathway, *SOD2,* encodes information for manganese superoxide dismutase (MnSOD) that neutralizes reactive superoxides. On the other hand, *MPO*, *CYBA*, and *NOS3* are part of pro-oxidant enzymes. They encode myeloperoxidase, the p22phox subunit of the NADPH oxidase (NOX), and endothelial nitric oxide synthase (eNOS), respectively. Myeloperoxidase released from leukocytes could oxidize LDL particles; the NOX enzyme is the major superoxide generator in the endothelial cells and VSMCs, whereas eNOS produces NO, a potential source for reactive nitrogen species. Polymorphisms involving these genes may be associated with CHD development in T2D. Initially, a study by Katakami et al., (2010) among 3819 T2D Japanese patients did not find significant differences in the allelic nor genotypic frequencies of these genes individually that associated with CHD [51]. However, subjects harboring several of the gene variants had a significant increase in CHD risk, indicating that their effects, although small, could act synergistically and influence disease development. In this population, the lowest MI prevalence was seen among subjects carrying ≤3 risk alleles (2%), while the highest prevalence was observed in those carrying 8–10 risk alleles (8.5%) (*p*_trend_ = 0.018) [51]. Apart from that, the study also reported that CHD risk in this population correspondingly rose with an increasing number of risk alleles. By taking ≤4 risk alleles as a reference, a combination of 5–7 risk alleles marginally raised CHD risk by 1.70 times (*p* = 0.081), while a mixture of ≥8 risk alleles substantially increased CHD risk by 2.43 times (*p* = 0.029) [51].

The apo-A5 and PON-1 proteins, encoded by respective genes of the same name, are present on the HDL surface. They appear to take part in promoting LDL metabolism and protecting it against oxidation, as they are related to energy and lipoprotein metabolisms as well as antioxidant mechanisms, respectively [74,157]. Among T2D Indian participants, the –1131T/C variant of *APOA5* and the Q192R variant of *PON1* increased CHD risk by 1.5 times separately, with both effects having a similar strength of association (OR*_APOA5_*: 1.50, *p* = 0.034; OR*_PON1_*: 1.49, *p* = 0.023). The interaction between these genes markedly enhanced CHD risk to more than 4-fold compared to their original effect sizes, indicating a synergistic effect (OR: 4.38, *p* = 0.038). However, only the TC heterozygotes of –1131T/C and the RR homozygotes of Q192R exhibited significant interactions in this population [22].

### 3.2. SNP-SNP Interaction in a Single Gene

Single nucleotide polymorphisms (SNP) of a particular gene may or may not alter the function of its protein product. However, carrying several different SNPs for that particular gene may eventually affect protein activity. An SNP–SNP interaction refers to the collaborative influences of having several SNPs of a gene that may influence disease development.

Several studies reported the SNPs interaction involving the adiponectin gene, *ADIPOQ*, which is related to energy and lipoprotein metabolisms, and their effects on CHD development, especially in diabetic patients. Among 241 T2D Iranian patients, Esteghamati et al., (2012) observed that the *ADIPOQ* 276T polymorphism was significantly associated with a 0.39-times decreased CHD risk (Table 3) [13].

Despite the T45G variant not exhibiting any impact on CHD (effect size not reported), its haplotypic combination with G276T slightly decreased the strength of association (i.e., the *p*-values) for the protective effect of 276T against CHD [13]. Individuals carrying 45T/276T and 45G/276T haplotypes had 0.47 times (*p* = 0.03) and 0.33 times (*p* = 0.02) decreased CHD risk, respectively, compared to the original individual effect of 276T (OR: 0.39, *p* = 0.001) [13]. Correspondingly, Mohammadzadeh et al. (2016) also found significant SNPs interaction involving the same *ADIPOQ* G276T and T45G polymorphisms in a separate study. The individual assessment of 45G and 276G allelic distribution did not yield a significant association with CHD among Iranian T2D individuals, with their respective ORs at 0.59 (*p* = 0.185) and 0.58 (*p* = 0.086) [65]. Meanwhile, the SNP–SNP interaction of 45G and 276G substantially reduced the risk for CHD by 0.37 times (*p* = 0.022) [65]. On the other hand, Tong et al. (2013) reported that one out of eleven *ADIPOQ* SNPs (rs266729*C/G) had a significant association with CHD in T2D Chinese patients for its G risk allele (OR: 1.64, *p* = 0.00095) [58]. Analysis of SNP–SNP interactions using a haplotypes combination of three *ADIPOQ* SNPs (rs266729*C/G, rs182052*G/A, and rs1501299*G/T) showed significantly higher CHD risk for CGG/GAG (OR: 2.13, *p* = 0.001) and CAG/GAG (OR: 2.26, *p* = 0.005) [58]. However, the GGG/GAG combination recorded the highest CHD risk of more than 3-fold (OR: 3.39, *p* = 0.0001). The selection of these SNPs was based on their significant association with serum adiponectin levels in this study population [58].

The adiponectin receptor gene (*ADIPOR1*), also related to energy and lipoprotein metabolisms, had significant SNP interactions that modified the gene influence towards CHD development among T2D. As demonstrated by Jin et al., 2014, the individual effect of rs3737884*G and rs16850797*C conferred heightened CHD risk in Han Chinese T2D patients by 2.42- and 1.71-times, respectively, in additive models [63]. Combinations of two risk alleles from either one of the two SNPs produced a similar effect with an OR of 2.44 (*p* = 0.002), while the presence of three and more risk alleles further increased the risk to 3.38 times (*p* = 1.14 × 10^−5^) [63]. Meanwhile, in a study by Wang et al. (2016), three individual polymorphisms markedly associated with increased CHD risk were reported at 1.84 times (*p* = 6.54 × 10^−6^) for rs3737884*A/G, 1.57 times (*p* = 0.001) for rs16850797*G/C, as well as 1.70 times (*p* = 0.002) for rs7514221*T/C in a T2D Chinese population [67]. A commonly occurring haplotype with a combination of GCT alleles from the respective polymorphisms did not result in a higher CHD risk than their individual effects (OR: 1.61, *p* = 8.74 × 10^−4^) [67]. However, the SNP interaction in other commonly occurring haplotypes (AGT) appeared to confer protection against CHD (OR: 0.49, *p* = 1.10 × 10^−6^) [67].

The SNP interaction involving the *NOS3* gene, which is related to vascular and endothelial pathology, also had been reported in T2D Asians with CHD. Among three *NOS3* gene polymorphisms tested by Narne et al. (2013) in Indian T2D patients, only the C allele carriers of –786T/C had significantly increased CHD risk (OR: 1.84, *p* = 0.004), but not for the *NOS3* 4b carriers of the intron 4a/b variant (OR: 0.98, *p* = 1.00) nor T allele carriers of G894T variants (OR: 1.35, *p* = 0.19) [59]. Interaction among the three SNPs revealed that the TbG haplotype had the highest frequency in T2D in this population. The combined TbG SNP also showed a protective effect against CHD in T2D patients, with odds of 0.68 (*p* = 0.03), compared to non-TbG haplotypes [59].

Analysis of the allelic frequencies of four variants in the *PCSK1* gene, which is related to hormonal regulation of glucose metabolism by the GLP-1 incretin, did not yield a significant association of CHD in T2D Chinese patients except for one, the rs3811951. The odds of having CHD were 0.85 (*p* = 0.17) for rs6234*C/G, 1.11 (*p* = 0.44) for rs6233*T/C, 1.21 (*p* = 0.09) for rs156019*T/A, and 0.75 (*p* = 0.01) for rs3811951*A/G [61]. Conversely, genotypes for the last two polymorphisms had a significant association with CHD, wherein the additive and dominant models for rs156019 increased the risk, while the recessive model for rs3811951 decreased CHD risk. Meanwhile, analysis of the SNP interaction showed that the CTAG combination from each of the four polymorphisms was the second most common haplotype after GTTA present in this population. GTTA did not have any association with CHD, but CTAG was associated with a significantly decreased CHD risk, with odds of 0.69 (*p* = 0.02) [61].

### 3.3. Gene-Environment Interaction

Gene-environment interaction is a condition where the effect of environmental exposure modifies the genetic effect on the risk of a disease [246]. Gene-environment interaction may also influence the disease’s phenotype. Although the term usually refers to the difference in temperature, exposure to pollutants, and sociodemographic characteristics, other factors such as age, gender, smoking habits, and biophysical parameters have been included and regarded as environmental factors as well. In cardiometabolic and nutrigenic research, environmental factors are also comprised of physical inactivity, dietary and sleep patterns, as well as other lifestyle choices [247].

The effects on CHD among T2D Chinese subjects brought by a member of the kinesin-9 superfamily, *KIF6* gene, that is related to cytoskeletal function and intracellular transport appeared to be modified by gender. Although *KIF6* 719Trp/Arg + Arg/Arg heterozygotes had almost 2-fold higher CHD risk, this association was not significant statistically (OR: 1.70, *p* = 0.4083) (Table 4) [17]. A subgroup analysis among female T2D subjects obtained a similar result, with the odds of having CHD slightly less than one (OR: 0.99, *p* = 0.9582). On the other hand, male gender interacted with the *KIF6* gene, whereby male carriers of 719Trp/Arg + Arg/Arg had a 5-fold increased CHD risk compared to their 719Trp/Trp counterparts (OR: 5.21, *p <* 0.01). Likewise, higher serum TG levels in CHD + T2D were observed only among men with the risk genotype but not among females or overall patients [17].

Several genes could interact with T2D in causing CHD among Asians, including the *ENPP1*, *AP2A2*, and 9p21 locus. These genes are related to the hormonal regulation of glucose metabolism, energy and lipoprotein metabolisms, as well as cell cycle regulation, respectively. As discussed earlier, the ENPP-1 protein is a regulator of the insulin signaling pathway. While Chinese patients with AC + CC genotypes of the *ENPP1* rs1044498 polymorphism suffered a 4-fold higher CHD risk (OR: 4.15, *p <* 0.01), those with T2D co-morbidity had a staggering 12-fold higher risk compared to the AA genotypes (OR: 12.81, *p <* 0.01), suggesting an *ENPP1*–T2D interaction [69]. For the *AP2A2* gene, the risk for CHD was conferred by the T allele of the rs7396366 polymorphism, while the G allele provided a protective effect. As such, the rs7396366*GG homozygotes had a significantly reduced risk for CHD (OR: 0.68, *p* = 0.042) that did not become modified even with a positive T2D status (OR: 0.91, *p* = 0.748). Individuals with the GT + TT risk genotypes of the *AP2A2* rs7396366 variant alone did not have increased CHD risk (OR: 1.13, *p* = 0.545). In contrast, the rs7396366 risk genotypes and T2D interaction significantly elevated CHD risk by more than 2-fold (OR: 2.33, *p* = 0.009) [73]. In North American populations, the interaction between the 9p21 locus and T2D associated with a CHD outcome was well-documented. In a prospective cohort study, T2D patients carrying the 9p21 locus risk allele with poor glycemic control at baseline had a stronger association with future CHD development [248], while in a case-control study, the 9p21 locus was associated with increased CHD severity [249]. A study among the Asian Chinese reported similar findings. The G allele of 9p21 locus rs10757274 polymorphism was a risk allele in this population. Considering the rs10757274*AA genotype as a reference, the CHD risk in non-diabetics was 1.60 times higher among AG + GG genotypes. The concurrent presence of T2D substantially tripled the CHD risk in rs10757274*AG + GG genotypes from 1.60 times (*p* = 0.0329) to 4.38 times (*p* = 0.0001) [62]. However, T2D status did not appear to mediate any change of CHD risk among the rs10757274*AA genotypes (OR: 1.68, *p* = 0.0943) [62].

The *PRKAA1* gene stores the instruction for AMPK-α1 protein synthesis. As discussed earlier, AMPK carries the adiponectin signal, resulting in the prevention of endothelial dysfunction and atherosclerosis, apart from its function in energy metabolism [109,136]. Ma et al., (2014) observed that T2D Chinese patients harboring the C allele of *PRKAA1* rs3805489 had decreased CHD risk in allelic and dominant inheritance models [60]. They also observed a significant gene–environment interaction between *PRKAA1* and smoking. A positive history of cigarette smoking, either as a current or ever smoker, did not affect the protective effects of the rs3805489*C allele but modified the effect of the A risk allele. Considering “never smoked + AC/CC” individuals as the reference, the “ever smoked + AC/CC” did not have a significant association with CHD risk (OR: 1.18, *p* = 0.664) [60]. Although the “never smoked + AA” subjects did not have a strong association with increased CHD risk despite carrying the A risk allele (OR: 0.96, *p* = 0.895), the interaction between the *PRKAA1* risk variant and smoking history in the “ever smoked + AA” group substantially enhanced the risk by 3-fold (OR: 3.02, *p* = 0.005) [60].

Another gene–environment interaction linked to increased CHD risk has been reported for the *APOE* gene, which is related to energy and lipoprotein metabolisms. As mentioned in the previous section, the E3 allele of *APOE* conferred protection against CHD due to its association with higher antioxidant proteins, higher HDL, and lower LDL levels. Although the E4 allele increased CHD risk in an Asian population, the absence of other environmental factors caused the risk to be negligible. Considering *APOE* E3/E3 as a reference, the CHD risk for E3/E4 carriers was only 1.02 times non-significantly higher (*p* = 0.970). With the presence of either smoking or overweight/obesity, the CHD risk among E3/E3 carriers markedly increased by 2-fold (OR: 2.24, *p* = 0.018). Genetic interaction with either smoking or overweight/obesity among the E3/E4 carriers was more pronounced compared to E3/E3, with a staggering 10-fold higher CHD risk (OR: 10.48, *p <* 0.0001) compared to 1.02 times in those without the environmental risk factors [18].

For the *UBE2Z* gene related to antioxidant mechanisms, the rs46522*TT genotype was associated with higher CHD risk among T2D Chinese patients, as previously mentioned (OR: 1.28, *p* = 0.020). When analyzed as a continuous variable, there was a positive interaction between BMI and *UBE2Z* rs46522 polymorphism, wherein, with every 1-unit of BMI, CHD risk increased by 0.012 (β = 0.012, *p* = 0.028) [70]. As a categorical variable, subgroup analysis among overweight/obese Chinese T2D patients harboring the rs46522*TT genotype increased CHD risk from 1.28 times to 1.54 times (*p* = 0.018) [70]. Like *APOE*, the *UBE2Z* also interacts with smoking and raises the probability of CHD in T2D. Taking the rs46522*CT + CC genotype as a reference, non-smoker TT homozygotes did not have increased CHD risk (OR: 0.89, *p* = 0.611). In contrast, being a smoker substantially elevated the risk of having CHD in both rs46522CT + CC and TT genotypes by 1.67 times (*p* = 0.019) and 3.00 times (*p <* 0.001), respectively [70].

Concurrent overweight/obesity and hypertension could modify the activity of the adiponectin gene, *ADIPOQ*, a gene that is related to energy and lipoprotein metabolism, and influence disease development. In a Japanese T2D population, the G risk allele of the *ADIPOQ* G276T variant heightened CHD risk. As mentioned before, the odds of having CHD were 1.66 times among T2D 276GG homozygotes (*p* = 0.0098). Notably, the overweight/obesity status barely but significantly modified the CHD risk (OR: 1.67, *p* = 0.0090), while concurrent hypertension did not produce any effects (OR: 1.25, *p >* 0.05) [56]. In comparison, T2D subjects carrying the heterozygous protective 276T allele did not have heightened CHD risk even when they were obese (OR: 1.17, *p >* 0.05). However, high blood pressure appeared to modify the 276T protective allele effects, whereby hypertensive subjects with 276GT + TT genotypes had 2.33-times increased risk for CHD (*p* = 0.0095) [56].

The CHD risk conferred by *HDAC9*, a gene related to the regulation of gene expression and cell signaling, could be modified by environmental risk factors, including T2D, hyperlipidemia, and overweight/obesity. In a Chinese population, the gene rs2107595*A polymorphism had a significant association with CHD in both the general population and the T2D sub-population. Using the multifactor dimensionality reduction approach, Wang et al., (2016) reported a significant gene–environment interaction concerning *HDAC9*. Individually, the rs2107595 variant, T2D status, overweight/obese BMI category, as well as hyperlipidemia exerted respective 0.30%, 0.58%, 0.36%, and 0.31% impacts on CHD, independently [66]. However, these effects were particularly higher when rs2107595 interacted with T2D (3.66%) and BMI (1.10%). The interaction between rs2107595 and hyperlipidemia did not produce an impact on CHD additional to hyperlipidemia alone (0.31%) [66].

As described earlier, smoking, alcohol intake, and T2D are modifiable non-genetic risk factors for CHD in the Asian populations. In a study among 3781 Chinese participants, they are associated with higher CHD risk with statistical significance achieved for smoking (OR: 1.37, *p <* 0.05) and alcohol intake (OR: 1.33, *p <* 0.05), but not for T2D (OR: 1.10, *p >* 0.05). However, a combination of smoking and alcohol intake slightly but significantly raised the CHD risk 1.64-fold (*p <* 0.05), while the addition of T2D further increased the risk 1.83-fold (*p <* 0.05). Meanwhile, a general Asian population with *TXNIP* rs7212*CG + GG genotypes had 1.26 times higher CHD risk. The antioxidant function of the *TXNIP* gene has been described in an earlier section. Interactions between rs7212*CG + GG risk genotypes with smoking, alcohol intake, and T2D markedly tripled CHD risk in this population (OR: 3.70, *p <* 0.05) compared to the genetic and environmental factors and individual effects [68].

Environmental interaction could also change the effect direction of a gene in disease development. For instance, the G allele of the *PARP1* rs1136410, which is related to DNA damage repair, exerted a significant protective effect against CHD among Chinese subjects, with the GG genotypes having 0.73 times lesser risk compared to non-carriers [71]. The genetic interaction of *PARP1* with smoking and hyperlipidemia status significantly increased the odds of having CHD, although these were still within the protective range (OR_smoking+GG_: 0.94, *p* = 0.031; OR_hyperlipidemia+GG_: 0.96, *p* = 0.025). On the contrary, *PARP1* interaction with T2D pushed the odds towards the non-protective side, although the risk was minimal (OR: 1.02, *p* = 0.01) [71].

## 4. Perspectives and Concluding Remarks

The genes included in this manuscript were collected by electronically searching for papers that assessed the genes’ associations with CHD in T2D Asians in order to address our main objectives. These include (i) reviewing the current evidence of genetic risk factors for CHD, (ii) summarizing the proposed mechanisms of these genes and how they may be associated with increased CHD risk, and (iii) reviewing the gene–gene and gene–environment interactions in CHD patients among T2D Asian populations. We used the PubMed database as our main search strategy using relaxed keyword combinations of “type 2 diabetes”, “coronary heart disease”, “coronary artery disease”, “myocardial infarction”, “ischemic heart disease”, “acute coronary syndrome”, “mutation”, “variant”, and “polymorphism”, with a similar search performed in Google Scholar. The reference sections of selected articles were also screened for potential papers. Article types were restricted to meta-analysis and original research on human subjects. We broadly defined Asian populations as the local people residing within the Asian continent or those with an Asian ancestry background. Additionally, papers describing the molecular mechanisms of each gene were sought. For this purpose, we did not restrict article types that included in vitro and in vivo studies in order to provide sufficient depth of information.

The majority of these papers used the candidate gene while others utilized GWA study designs. Some of the genetic variations were only examined in terms of their associations with CHD in the general population without further analysis among T2D individuals. Since T2D is a significant risk for CHD, its severity, as well as CHD-death, and genetic variations have been shown to interact with T2D status, duration, and HbA_1c_ levels, it is particularly important to include this secondary analysis among the T2D subset when performing genetic association studies of CHD. Overall, eight SNPs showed significant associations with CHD exclusively in T2D Asians but not T2D Caucasians (i.e., *APOE* E4, *APOA5* –1131C, *ADRB3* 64Arg, *BChE*-K variant, *ADIPOQ* –11377G, *ADIPOQ* 45G, *UBE2Z* rs46522*T, and 9p21 rs10757274*G), five SNPs showed a similar association in both populations (i.e., *PON1* 192R, *PON2* 311Cys, 9p21 rs10811661*T, 9p21 rs944801*C, and *ENPP1* rs1044498*C), one SNP had a reversed association with CHD in T2D Caucasians (i.e., *ADIPOQ* 276T), twelve SNPs had not been tested for CHD among T2D Caucasians (i.e., *PRKAA1* rs3805489*A, *AP2A2* rs7396366*T, *SCYA11* 23Ala, *NOS3* –786C, *NOS3* 4b, *TXNIP* rs7212*G, 9p21 rs10757283*C, *PARP1* rs1136410*G, *PCSK1* rs156019*T, *PCSK1* rs3811951*A, *KIF6* 719Arg, and *HDAC9* rs2107595*A), while different SNPs of the *ADIPOR1* gene were associated with CHD in T2D Asians (i.e., rs3737884*G, rs16850797*C, and rs7514221*C) and T2D Caucasians (i.e., rs7539542*G and rs10920531*A).

One of the utilities of genetic risk identification of CHD is in personalized medicine, in which a genetic risk score can be calculated to predict the likelihood of future CHD events. Previous studies that aimed at predicting T2D and CHD using various genetic variants constructed their polygenic risk score (PRS) using different approaches. For instance, Reisberg et al. (2017) used 49,310 SNPs to predict CHD and 7502 SNPs to predict T2D that were derived from the European populations [250]. The study team tested the PRS on different ethnic groups who participated in the 1000 Genomes Project, including East Asians, South Asians, Europeans, Americans, and Africans [250]. The study concluded that a PRS built from one population could not readily be applied to other populations due to differences in their genetic makeup, predisposing one to the systematic error of risk estimates [250]. A recently published systematic review by Padilla-Martínez et al. (2020) [251] shed further light on this matter. Between 2006 and 2018, fourteen published papers that attempted to predict T2D constructed their PRS from as little as three to as many as seven million SNPs [251]. Therefore, at present, we do not know how many mutations are required to develop T2D in Asians compared to Western populations. There is no T2D animal model that concurrently has all the genetic alterations as depicted in this paper that can be used to justify CHD development reflecting Asian populations as well.

The list of genetic and environmental factors in this paper is non-exhaustive and restricted only to those with evidence of gene–gene and gene–environment interactions that could modify CHD risk in the T2D Asian populations. Many of the genes’ mechanisms contributing to the disease are still not well-elucidated, serving as potential prospects for future research, either to further enhance the molecular understanding of CHD in T2D or as novel therapeutic targets. These genetic factors, along with gene–gene and gene–environment interactions, exemplify the complexity of CHD, particularly in the T2D Asian populations.

## Figures and Tables

**Table 1:** Genetic factors associated with coronary heart disease in type 2 diabetic Asian populations.

Source	Population/Sample Size	Country/Region	Genes/Loci	CHD Association		Allele	Model	CHD Risk, OR or HR (95% CI)	*p*-Value *
				Yes	No				
Zhou et al., 2009 [49]	194 CHD and 90 healthy controls	China	*PON1* L55M (rs854560)		✓				
Vaisi-Raygani et al., 2010 [41]	118 T2D, 162 CHD, 172 T2D + CHD, and 179 healthy controls	Iran	*BCHE*-K^ G1615A (likely rs1803274)	✓		A	Allelic	OR: 2.02 (1.40–3.10)	0.001
			*APOE^* (rs7412 and rs429358)	✓		E4	Recessive	OR: 7.80 (1.70–3.60)	0.008
Wang W. et al., 2010 [50]	2046 CHD	China	9p21.3 (rs1333049)		✓				
Wang Y. et al., 2010 [21]	1297 T2D	China	*SCYA11* Ala23Thr (rs1129844)	✓		Ala	Recessive	HR: 1.70 (1.10–2.61)	0.016
			*PON2* Ser311Cys (rs7493)	✓		Cys	Additive	HR: 1.42 (1.08–1.88)	0.013
			*ADRB3^* Trp64Arg (rs4994)	✓		Arg	Recessive	HR: 3.84 (1.18–12.50)	0.025
Katakami et al., 2010 [51]	3819 T2D	Japanese	*GCLM* -588C/T (rs17883901)		✓				
			*SOD2* Val16Ala (rs4880)		✓				
			*NOS3* G894T (rs1799983)		✓				
			*CYBA* C242T (rs4673)		✓				
			*MPO*-463G/A (rs2333227)		✓				
Bae et al., 2010 [52]	192 CHD (54 T2D) and 196 non-CHD controls (35 T2D)	Korea	*eNOS*-786T/C (rs2070744)	✓		C	Dominant	OR: 4.39 (1.80–10.71)	<0.050
			*eNOS* 4a/4b (rs61722009)	✓		4a	Dominant	OR: 4.20 (1.73–10.16)	<0.050
			*eNOS* 894G/T (rs1799983)		✓				
Bhaskar et al., 2011 [22]	250 CAD (160 T2D), 150 T2D, and 120 healthy controls	India	*PON1* Q192R (rs662)	✓		R	Allelic	OR: 1.49 (1.04–2.12)	0.023
			*APOA5^*−1131T/C (rs662799)	✓		C	Allelic	OR: 1.50 (1.01–2.22)	0.034
Cheng et al., 2011 [53]	Central China: 379 T2D, 496 CHD, and 849 healthy controls Northern China: 597 CHD and 846 healthy controls	China	9p21.3 (rs2383208)		✓				
			9p21.3 (rs10811661)	✓		T	Allelic	OR: 1.19 (1.06–1.33)	0.004
			9p21.3 (rs10757283)	✓		C	Allelic	OR: 1.18 (1.06–1.32)	0.001
Ergun et al., 2011 [54]	171 T2D and 80 healthy controls	Turkey	*PON1* L55M (rs854560)		✓				
			*PON1* Q192R (rs662)		✓				
Chaudhary et al., 2012 [18]	147 T2D + CHD, 155 T2D, and 149 healthy controls	Thailand	*APOE^* (rs7412 and rs429358)	✓		E4	Allelic	OR: 2.32 (1.17–4.61)	0.016
Esteghamati et al., 2012 [13]	114 T2D + CHD and 127 T2D	Iran	*ADIPOQ* T45G (rs2241766)		✓				
			*ADIPOQ* G276T (rs1501299)	✓		T	Additive	OR: 0.39 (0.22–0.68)	0.001
Ho et al., 2012 [55]	1417 T2D	Hong Kong	*PPARG* (rs1801282 G/C)	✓		C	Recessive	HR: 4.38 (1.03–18.57)	0.045
			*ADIPOQ* (rs1063539 C/G)		✓				
			*HNF4A* (rs1884614 T/C)		✓				
Katakami et al. 2012 [56]	2637 T2D	Japanese	*ADIPOQ* G276T (rs1501299)	✓		G	Recessive	OR: 1.66 (1.13–2.43)	0.009
Saini et al., 2012 [57]	28 T2D + CHD, 32 CHD, and 50 healthy controls	India	*eNOS* Glu298Asp (rs1799983)		✓				
Tong et al., 2013 [58]	560 T2D + CHD and 550 T2D	China	*ADIPOQ*−11377C/G (rs266729)	✓		G	Recessive	OR: 2.18 (1.32–3.46)	0.001
Narne et al., 2013 [59]	160 T2D + CHD and 121 T2D	India	*eNOS* -786T/C (rs2070744)	✓		G	Dominant	OR: 1.81 (1.05–3.13)	0.030
			*eNOS* intron 4a/b (rs61722009)		✓				
			*eNOS* G894T (rs1799983)		✓				
Ma et al., 2014 [60]	260 T2D + CHD and 144 T2D control	China	*PRKAA1* (rs3805489)	✓		C	Allelic	OR: 0.67 (0.48–0.92)	0.015
Wei et al., 2014 [61]	425 T2D + CHD and 258 T2D control	China	*PCSK1* (rs6230*T/C)		✓				
			*PCSK1* (rs6233*T/C)		✓				
			*PCSK1* (rs6234*C/G)		✓				
			*PCSK1* (rs156019*T/A)	✓		T	Additive	OR: 1.92 (1.23–3.00)	0.04
			*PCSK1* (rs3811951*A/G)	✓		G	Recessive	OR: 0.43 (0.24–0.77)	0.004
Wu et al., 2014 [17]	288 T2D + CHD, 312 T2D, and 346 healthy controls	China	*KIF6* Trp719Arg (rs20455)	✓		Arg	Dominant	OR: 5.21 (1.01–27.01)	<0.01
Zhang et al., 2014 [62]	502 MI (194 T2D) and 308 angiographic normal (75 T2D) controls	China	9p21 (rs10757274) ^	✓		G	Dominant	OR: 4.38 (2.56–7.47)	<0.0001
			6p24 (rs6903956)		✓				
Jin et al., 2014 [63]	165 T2D, 173 CHD, 174 T2D + CHD, and 145 healthy controls	China	*ADIPOR1* (rs7539542)		✓				
			*ADIPOR1* (rs3737884)	✓		G	Additive	OR: 2.42 (1.51–3.89)	2.49 × 10^−4^
			*ADIPOR1* (rs1342387)		✓				
			*ADIPOR1* (rs16850797)	✓		C	Additive	OR: 1.71 (1.11–2.62)	0.014
			*ADIPOR1* (rs12045862)		✓				
			*ADIPOR1* (rs7514221)		✓				
Sapkota et al., 2015 [42]	1956 T2D (723 CHD), 1608 non-T2D (1212 non-CHD)	US (Indian ancestry)	*APOE* (rs7412 and rs429358)		✓				
Mofarrah et al., 2016 [64]	152 T2D + angiographic CHD and 72 T2D controls	Iran	*ADIPOQ* T45G (rs2241766*T/G) ^	✓		G	Dominant	OR: 7.21 (2.02–25.73)	0.002
			*KALRN* (rs9289231*T/G)	✓		G	Dominant	OR: 5.02 (1.07–23.70)	0.041
			*FTO* (rs9939609*A/T)		✓				
Mohammadzadeh et al., 2016 [65]	100 T2D + CHD and 100 T2D controls	Iran	*ADIPOQ* T45G (rs2241766)		✓				
			*ADIPOQ* G276T (rs1501299)	✓		T	Additive	OR: 5.16 (1.02–26.18)	0.048
Wang et al., 2016 [66]	2317 CAD and 2404 healthy controls	China	*HDAC9* (rs2107595*G/A)	✓		A	Dominant	OR: 1.23 (1.09–1.39)	0.001
Wang F. et al., 2016 [67]	295 T2D, 316 CHD, 302 T2D + CHD, and 268 healthy controls	China	*ADIPOR1* (rs7539542)		✓				
			*ADIPOR1* (rs3737884)	✓		G	Dominant	OR: 2.69 (1.43–5.07)	0.002
			*ADIPOR1* (rs1342387)		✓				
			*ADIPOR1* (rs16850797)	✓		C	Dominant	OR: 1.44 (1.03–1.99)	0.032
			*ADIPOR1* (rs12045862)		✓				
			*ADIPOR1* (rs7514221)	✓		C	Dominant	OR: 1.75 (1.19–2.56)	0.004
Wang X. et al., 2016 [68]	595 T2D + CHD and 519 T2D	China	*TXNIP* (rs7212)	✓		G	Dominant	OR: 1.53 (1.18–1.99)	0.022
			*TXNIP* (rs7211)		✓				
			*TXNIP* (rs9245)		✓				
Sumi et al., 2017 [69]	198 CHD, 284 T2D + CHD, 160 T2D, and 271 healthy controls	India	*ENPP1* K121Q (rs1044498)	✓		C	Dominant	OR: 12.8 (4.97–37.18)	<0.01
Lu et al., 2017 [70]	390 T2D + CHD and 275 T2D	China	*UBE2Z ^* (rs46522)	✓		T	Additive	OR: 1.67 (1.09–2.57)	0.019
Wang et al., 2017 [71]	903 T2D + CAD and 726 T2D	China	*PARP1* (rs1136410)	✓		G	Recessive	OR: 1.02 (0.76–1.35)	0.010
Zhao et al., 2017 [72]	265,678 T2D and 260,365 CHD	Multiple countries including the Eastern and Southern Asian ancestries	*TCF7L2* (rs7903146)	✓		T	Allelic	OR: 1.04 (1.02–1.05)	2.6 × 10^−212^
			*HNF1A* I27L (rs1169288)	✓		A	Allelic	OR: 1.04 (1.03–1.06)	2.0 × 10^−12^
			*CTRB1/2* (rs7202877)	✓		T	Allelic	OR: 1.06 (1.04–1.09)	1.0 × 10^−8^
			*MRAS* (rs2306374)	✓		C	Allelic	OR: 1.06 (1.04–1.08)	9.8 × 10^−9^
			*ZC3HC1* R342H (rs11556924)	✓		C	Allelic	OR: 1.08 (1.06–1.10)	1.4 × 10^−19^
			*MIR17HG* (rs7985179)	✓		A	Allelic	OR: 1.05 (1.02–1.08)	1.5 × 10^−9^
			*CCDC92* (rs825476)	✓		T	Allelic	OR: 1.03 (1.02–1.05)	2.7 × 10^−9^
			*APOE^* (rs4420638)	✓		A	Allelic	OR: 0.89 (0.85–0.93)	2.6 × 10^−13^
Wang et al., 2018 [73]	335 CHD and 372 non-CHD	China	*AP2A2* (rs7396366)	✓		T	Dominant	OR: 2.33 (1.24–4.38)	0.009
			*BZRAP1* (rs2526378)		✓				

* Indicates significant *p*-value. ^ Significant association with CHD exclusive in T2D Asians but not T2D Caucasians. Symbol: (✓) Genes associated with CHD. Abbreviations: Ala (alanine), Arg (arginine), CAD (coronary artery disease), CHD (coronary heart disease), CI (confidence interval), Cys (cysteine), HR (hazard ratio), OR (odds ratio), Ser (serine), T2D (type 2 diabetes mellitus), Thr (threonine), Trp (tryptophan), Val (valine).

**Table 2:** Gene-gene interactions and their associations with CHD in T2D Asians.

Source	Population/ Sample Size	Country/ Region	Genes/Loci	Gene-Gene Interaction		CHD Risk, OR or HR (95% CI)	*p*-Value	Other Information
				Yes	No			
Vaisi-Raygani et al., 2010 [41]	118 T2D, 162 CHD, 172 T2D + CHD, and 179 healthy controls	Iran	*BCHE*-K G1615A (likely rs1803274) *APOE4* (rs7412 and rs429358)	✓		*BCHE*-K only: OR: 2.10 (1.30–3.60)	0.003 *	The presence of both *BCHE*-K and *APOE4* variants was significantly associated with a higher LDL, TG, and TC, and lower HDL.
						*APOE4* only: OR: 2.10 (1.21–4.45)	0.022 *	
						*BCHE*-K and *APOE4*: OR: 4.50 (1.40–14.50)	0.011 *	
Wang Y. et al., 2010 [21]	1297 T2D	China	*SCYA11* Ala23Thr (rs1129844) *PON2* Ser311Cys (rs7493) *ADRB3* Trp64Arg (rs4994)	✓		≤1 risk allele: Ref.	-	
						2 risk alleles: HR: 1.99 (1.09–3.66)	0.026 *	
						3 risk alleles: HR: 2.74 (1.42–5.26)	0.003 *	
						4 risk alleles: HR: 4.11 (1.65–10.23)	0.002 *	
Katakami et al., 2010 [51]	3819 T2D	Japan	*GCLM* –588C/T (rs17883901) *SOD2* Val16Ala (rs4880) *NOS3* G894T (rs1799983) *CYBA* C242T (rs4673) *MPO*–463G/A (rs2333227)	✓		Individual polymorphism did not associate with higher CHD prevalence.		Prevalence of MI: ≤3 risk alleles (2.0%), 8–10 risk alleles (8.5%); (*p*_trend_ = 0.018).
						≤4 combined risk alleles: Ref.	-	
						5–7 combined risk alleles: OR: 1.70 (0.94–3.07)	0.081	
						≥8 combined risk alleles: OR: 2.43 (1.10–5.37)	0.029 *	
Bhaskar et al., 2011 [22]	250 CAD (160 T2D), 150 T2D, and 120 healthy controls	India	*PON1* Q192R (rs662) *APOA5* –1131T/C (rs662799)	✓		rs662 only: OR: 1.49 (1.04–2.12)	0.023 *	The only significant interaction was between rs662*RR homozygote and rs662799*TC heterozygote.
						rs662799 only: OR: 1.50 (1.01–2.22)	0.034 *	
						rs662 and rs662799: OR: 4.38 (1.08–17.71)	0.038 *	
Lei et al., 2012 [24]	538 T2D	China	*ACE* I/D (rs4646994) *AT2R* G1675A (rs1403543)		✓			
Ho et al., 2012 [55]	1417 T2D	Hong Kong	*PPARG* (rs1801282*G/C) *ADIPOQ* (rs1063539*C/G) *HNF4A* (rs1884614*T/C)		✓			

* Indicates significant *p*-value. Symbol: (✓) Gene–gene interaction in T2D + CHD. Abbreviations: CHD (coronary heart disease), CI (confidence interval), HDL (high-density lipoprotein), HR (hazard ratio), LDL (low-density lipoprotein), MI (myocardial infarction), OR (odds ratio), Ref. (reference), T2D (type 2 diabetes mellitus), TC (total cholesterol), TG (triglyceride).

**Table 3:** SNP–SNP interactions in a single gene and their associations with CHD in T2D Asians.

Source	Population /Sample Size	Country/ Region	Genes/Loci	SNP-SNP Interaction		CHD Risk, OR or HR (95% CI)	*p*-Value	Other Information
				Yes	No			
Esteghamati et al., 2012 [13]	114 T2D + CHD and 127 T2D	Iran	*ADIPOQ* T45G (rs2241766) *ADIPOQ* G276T (rs1501299)	✓		45G: OR and 95% CI not reported	NS	
						276T: OR: 0.39 (0.22–0.68)	0.001 *	
						TT haplotype: OR: 0.47 (0.32–0.94)	0.03 *	
						GT haplotype: OR: 0.33 (0.13–0.83)	0.02 *	
Tong et al., 2013 [58]	560 T2D + CHD and 550 T2D	China	*ADIPOQ* C/G (rs266729) *ADIPOQ* G/A (rs182052) *ADIPOQ* G/T (rs1501299)	✓		rs266729*G: OR: 1.64 (1.35–2.01)	9.5 × 10^−4^ *	Each polymorphism was also associated with lower adiponectin levels.
						rs182052*A: OR: 1.18 (0.98–1.52)	0.113	
						rs1501299*T: OR: 0.83 (0.67–1.03)	0.102	
						CGG/GAG diplotype: OR: 2.13 (1.40–3.60)	0.001 *	
						CAG/GAG diplotype: OR: 2.26 (1.40–4.10)	0.005 *	
						GGG/GAG diplotype: OR: 3.39 (1.75–6.50)	1 × 10^−4^ *	
Narne et al., 2013 [59]	160 T2D + CHD and 121 T2D	India	*eNOS* –786T/C (rs2070744) *eNOS* intron 4a/b (rs61722009) *eNOS* G894T (rs1799983)	✓		–786C: OR: 1.84 (1.22–2.76)	0.004 *	
						intron 4b: OR: 0.98 (0.63–1.54)	1.00	
						894T: OR: 1.35 (0.86–2.14)	0.19	
						TbG haplotype: OR: 0.68 (0.49–0.96)	0.03 *	
Wei et al., 2014 [61]	425 T2D + CHD and 258 T2D controls	China	*PCSK1* (rs6234*C/G) *PCSK1* (rs6233*T/C) *PCSK1* (rs156019*T/A) *PCSK1* (rs3811951*A/G)	✓		rs6234G: OR: 0.85 (0.67–1.07)	0.17	
						rs6233C: OR: 1.11 (0.86–1.42)	0.44	
						rs156019A: OR: 1.21 (0.97–1.52)	0.09	
						rs3811951G: OR: 0.75 (0.59–0.94)	0.01 *	
						CTAG haplotype: OR: 0.69 (0.54–0.88)	0.02 *	
Jin et al., 2014 [63]	165 T2D, 173 CHD, 174 T2D + CHD, and 145 healthy controls	China	*ADIPOR1* (rs3737884) *ADIPOR1* (rs16850797)	✓		≤1 risk allele: Ref.	-	These effects were reported for T2D + CHD.
						2 risk alleles: OR: 2.44 (1.38–4.31)	0.002 *	
						≥3 risk alleles: OR: 3.38 (1.95–5.87)	1.14 × 10^−5^ *	
Mohammadzadeh et al., 2016 [65]	100 T2D + CHD and 100 T2D controls	Iran	*ADIPOQ* T45G (rs2241766) *ADIPOQ* G276T (rs1501299)	✓		45G: OR: 0.59 (0.28–1.28)	0.1852	
						276G: OR: 0.58 (0.31–1.08)	0.0864	
						GG haplotype: OR: 0.37 (0.16–0.86)	0.022 *	
Wang F. et al., 2016 [67]	295 T2D, 316 CHD, 302 T2D + CHD, and 268 healthy controls	China	*ADIPOR1* (rs3737884) *ADIPOR1* (rs16850797) *ADIPOR1* (rs7514221)	✓		rs3737884*G: OR: 1.84 (1.41–2.41)	6.54 × 10^−6^ *	
						rs16850797*C: OR: 1.57 (1.22–2.03)	0.001 *	
						rs7514221*C: OR: 1.70 (1.22–2.38)	0.002 *	
						AGT haplotype: OR: 0.49 (0.37–0.65)	1.10 × 10^−6^ *	
						GCT haplotype: OR: 1.61 (1.21–2.13)	8.74 × 10^−4^ *	
Ma et al., 2017 [245]	159 T2D and 288 T2D + CHD	China	*STK11* (rs35369365) *STK11* (rs9282860) *STK11* (rs12977689)		✓			

* Indicates significant *p*-value. Symbol: (✓) SNP-to-SNP interaction in T2D + CHD. Abbreviations: CHD (coronary heart disease), CI (confidence interval), HR (hazard ratio), NS (not significant), OR (odds ratio), SNP (single nucleotide polymorphism), T2D (type 2 diabetes mellitus).

**Table 4:** Gene-environment interactions and their associations with CHD in T2D Asians.

Source	Population/ Sample Size	Country/Region	Genes/Loci	Environment	Gene-Environment Interaction		CHD Risk, OR or HR (95% CI)	*p*-Value	Other Information
					Yes	No			
Chaudhary et al., 2012 [18]	147 T2D + CHD, 155 T2D, and 149 healthy controls	Thailand	*APOE*	Smoking	✓		E3/E3 only: Ref.	-	
							E3/E3 + smoking/obesity: OR: 2.24 (1.15–4.35)	0.018 *	
				Obesity	✓		E3/E4 only: OR: 1.02 (0.34–3.06)	0.970	
							E3/E4 + smoking/obesity: OR: 10.48 (3.56–30.79)	<0.0001 *	
Esteghamati et al., 2012 [13]	114 T2D + CHD and 127 T2D	Iran	*ADIPOQ* G276T (rs1501299)	Age		✓			
				Gender		✓			
Katakami et al., 2012 [56]	2637 T2D	Japan	*ADIPOQ* G276T (rs1501299)	Obesity	✓				
				Gender		✓	276GG only: OR: 1.66 (1.13–2.43)	0.0098 *	
				Age		✓	276GT/TT + obesity: OR: 1.17 (0.73–1.89)	NS	
				Smoking		✓	276GG + obesity: OR: 1.67 (1.14–2.44)	0.0090 *	
				T2D years		✓	276GG + HTN: OR: 1.25 (0.80–1.93)	NS	
				HbA_1c_		✓	276GT/TT + HTN: OR: 2.33 (1.23–4.41)	0.0095 *	
				HTN	✓				
				Dyslipidemia		✓			
Ma et al., 2014 [60]	260 T2D + CHD and 144 T2D	China	*PRKAA1* (rs3805489)	Smoking	✓		AC/CC + never smoked: Ref.	-	
							AA + never smoked: OR: 0.96 (95% CI not reported)	0.895	
							AC/CC + ever smoked: OR: 1.18 (95% CI not reported)	0.664	
							AA + ever smoked: OR: 3.02 (1.39–6.57)	0.005 *	
Wu et al., 2014 [17]	288 T2D + CHD, 312 T2D, and 346 healthy controls	China	*KIF6* Trp719Arg (rs20455)	Gender	✓		719Trp/Arg + Arg/Arg: OR: 1.70 (0.71–7.30)	0.4083	Trp/Arg + Arg/Arg in male T2D + CHD is also associated with lower TG than healthy controls.
							719Trp/Arg + Arg/Arg in male: OR: 5.21 (1.01–27.01)	<0.01 *	
							719Trp/Arg + Arg/Arg in female: OR: 0.99 (0.56–1.72)	0.9582	
Zhang et al., 2014 [62]	502 MI and 308 angiographic normal controls	China	9p21 (rs10757274)	T2D	✓		AA: Ref.	-	
							GG/GA: OR: 1.60 (1.04–2.46)	0.0329 *	
							AA + T2D: OR: 1.68 (0.92–3.08)	0.0943	
							GG/GA + T2D: OR: 4.38 (2.56–7.47)	0.0001 *	
Wang et al., 2016 [66]	2317 CAD and 2404 healthy controls	China	*HDAC9* (rs2107595 *G/A)	T2D	✓		-	-	Using MDR: rs2107595 only (0.30%), T2D only (0.58%), BMI only (0.36%), hyperlipidemia only (0.31%), rs2107595 + T2D (3.66%), rs2107595 + hyperlipidemia (0.81%), and rs2107595 + BMI (1.10%).
				BMI	✓				
				Hyperlipidemia	✓				
Wang X. et al., 2016 [68]	1818 CHD and 1963 healthy controls	China	*TXNIP* (rs7212)				Smoking (1): OR: 1.37 (1.14–1.64)	<0.05 *	
							Alcohol (2): OR: 1.33 (1.10–1.61)	<0.05 *	
				Smoking			T2D (3): OR: 1.10 (0.89–1.35)	NS	
				Alcohol intake	✓		CG + GG (4): OR: 1.26 (1.10–1.46)	0.001 *	
				T2D			(1) + (2): OR: 1.64 (1.22–2.18)	<0.05 *	
							(1) + (2) + (3): OR: 1.83 (1.04–3.23)	<0.05 *	
							(1) + (2) + (3) + (4): OR: 3.70 (2.29–5.60)	<0.05 *	
Sumi et al., 2017 [69]	198 CHD, 284 T2D + CHD, 160 T2D, and 271 healthy controls	India	*ENPP1* K121Q (rs1044498)	T2D	✓		AC + CC: OR: 4.15 (2.61–6.73)	<0.01 *	
							AC + CC with T2D: OR: 12.81 (4.97–37.18)	<0.01 *	
Lu et al., 2017 [70]	390 T2D + CHD and 275 T2D	China	*UBE2Z* (rs46522)	BMI	✓		TT only: OR: 1.28 (1.04–1.57)	0.020 *	rs46522 with overweight/obesity increased CHD risk (β = 0.012, *p*_interaction_ = 0.028).
							TT + overweight/obesity: OR: 1.54 (1.08–2.19)	0.018 *	
Lu et al., 2017 [70]	390 T2D + CHD and 275 T2D	China	*UBE2Z* (rs46522)	Smoking	✓		TC/CC + non-smoker: Ref.	-	
							TT + non-smoker: OR: 0.89 (0.59–1.35)	0.611	
							TC/CC + smoker: OR: 1.67 (1.09–2.56)	0.019 *	
							TT + smoker: OR: 3.00 (1.88–4.79)	<0.001 *	
Wang et al., 2017 [71]	2803 CAD and 2840 healthy controls	China	*PARP1* (rs1136410)	Smoking	✓		GG only: OR: 0.73 (0.63–0.85)	6.45 × 10^−5^ *	
				Hyperlipidemia	✓		GG + smoking: OR: 0.94 (0.71–1.22)	0.031 *	
				T2D	✓		GG + hyperlipidemia: OR: 0.96 (0.72–1.28)	0.025 *	
							GG + T2D: OR: 1.02 (0.76–1.35)	0.01 *	
Wang et al., 2018 [73]	335 CHD and 372 non-CHD	China	*AP2A2* (rs7396366)	T2D	✓		GG only: OR: 0.68 (0.46–0.99)	0.042 *	
							GG + T2D: OR: 0.91 (0.50–1.64)	0.748	
							GT/TT only: OR: 1.13 (0.77–1.65)	0.545	
							GT/TT + T2D: OR: 2.33 (1.24–4.38)	0.009 *	

* Indicates significant *p*-value. Symbol: (✓) Gene-environment interaction in T2D + CHD. Abbreviations: Arg (arginine), BMI (body mass index), CAD (coronary artery disease), CI (confidence interval), CHD (coronary heart disease), HR (hazard ratio), HTN (hypertension), HbA_1c_ (glycated haemoglobin), MDR (multifactor dimensionality reduction), MI (myocardial infarction), NS (not significant), OR (odds ratio), Ref. (reference), T2D (type 2 diabetes mellitus), TG (triglyceride), Trp (tryptophan).

## Data Availability

Data that support the reported results can be found at PubMed (National Library of Medicine) and Google Scholar databases.
